# The BRAHMA-associated SWI/SNF chromatin remodeling complex controls *Arabidopsis* seed quality and physiology

**DOI:** 10.1093/plphys/kiae642

**Published:** 2024-12-11

**Authors:** Magdalena Wrona, Julia Zinsmeister, Michal Krzyszton, Claire Villette, Julie Zumsteg, Pierre Mercier, Martine Neveu, Sebastian P Sacharowski, Rafał Archacki, Boris Collet, Julia Buitink, Hubert Schaller, Szymon Swiezewski, Ruslan Yatusevich

**Affiliations:** Institute of Biochemistry and Biophysics PAS, Warsaw 02-106, Poland; Institute of Biochemistry and Biophysics PAS, Warsaw 02-106, Poland; Institute of Biochemistry and Biophysics PAS, Warsaw 02-106, Poland; Institut de Biologie Moléculaire des Plantes, Université de Strasbourg, Strasbourg 67084, France; Institut de Biologie Moléculaire des Plantes, Université de Strasbourg, Strasbourg 67084, France; Institut de Biologie Moléculaire des Plantes, Université de Strasbourg, Strasbourg 67084, France; INRAE, Institut Agro, Université d'Angers, IRHS, Angers 49000, France; Institute of Biochemistry and Biophysics PAS, Warsaw 02-106, Poland; Laboratory of Systems Biology, Faculty of Biology, University of Warsaw, Warsaw 02-096, Poland; Université Paris Saclay, INRAE, AgroParisTech, Institute Jean-Pierre Bourgin for Plant Sciences (IJPB), 78000 Versailles, France; INRAE, Institut Agro, Université d'Angers, IRHS, Angers 49000, France; Institut de Biologie Moléculaire des Plantes, Université de Strasbourg, Strasbourg 67084, France; Institute of Biochemistry and Biophysics PAS, Warsaw 02-106, Poland; Institute of Biochemistry and Biophysics PAS, Warsaw 02-106, Poland

## Abstract

The SWI/SNF (SWItch/Sucrose Non-Fermentable) chromatin remodeling complex is involved in various aspects of plant development and stress responses. Here, we investigated the role of BRM (BRAHMA), a core catalytic subunit of the SWI/SNF complex, in *Arabidopsis thaliana* seed biology. *brm-3* seeds exhibited enlarged size, reduced yield, increased longevity, and enhanced secondary dormancy, but did not show changes in primary dormancy or salt tolerance. Some of these phenotypes depended on the expression of *DOG1*, a key regulator of seed dormancy, as they were restored in the *brm-3 dog1-4* double mutant. Transcriptomic and metabolomic analyses revealed that BRM and DOG1 synergistically modulate the expression of numerous genes. Some of the changes observed in the *brm-3* mutant, including increased glutathione levels, depended on a functional *DOG1*. We demonstrated that the BRM-containing chromatin remodeling complex directly controls secondary dormancy through *DOG1* by binding and remodeling its 3′ region, where the promoter of the long noncoding RNA *asDOG1* is located. Our results suggest that BRM and DOG1 cooperate to control seed physiological properties and that BRM regulates *DOG1* expression through *asDOG1*. This study reveals chromatin remodeling at the *DOG1* locus as a molecular mechanism controlling the interplay between seed viability and dormancy.

## Teaser

A chromatin remodeling complex activates the expression of a noncoding transcript, suppressing the expression of a seed dormancy regulator *Delay of germination 1* gene.

## Introduction

Seeds encapsulate plant embryos in a state of suspended development, poised to resume life cycle upon encountering favorable environmental conditions. Many plant species, including *Arabidopsis thaliana*, produce seeds that can be stored in a dry stage for an extended time. This ability is known as seed longevity. In addition, seeds can also postpone germination despite optimal conditions, in a process known as dormancy that helps to adjust germination capability to changes in the environment. Dormancy established during seed maturation is known as primary dormancy and is defined as a state in which freshly harvested seeds cannot germinate even under favorable conditions. Primary dormancy can be relieved by different means including after-ripening—dry storage, or stratification—exposure to cold in the imbibed state. In contrast, dormancy developed by a nondormant, imbibed seed in response to an unfavorable germination condition is known as secondary dormancy (Cadman et al. 2006; Finch-Savage and Leubner-Metzger 2006; Baskin and Baskin 2014).

*DELAY OF GERMINATION1* gene (*DOG1*), the main regulator of seed dormancy, has been identified by population analysis (Bentsink et al. 2006) and further characterized in numerous molecular studies (Carrillo-Barral et al. 2020). Primary dormancy and longevity are both acquired during seed development and are strictly regulated by numerous external and internal factors (Holdsworth et al. 2008; Sano et al. 2016). Interestingly, in *Arabidopsis*, a tradeoff between seed longevity and dormancy was described, as deep dormancy was associated with low longevity, suggesting that longevity and dormancy are genetically negatively correlated. On the contrary, *DOG1* has been shown to act as a positive regulator of both dormancy and longevity as *dog1* mutants show low primary and secondary dormancy as well as low longevity (Nguyen et al. 2012; Dekkers et al. 2013; Footitt et al. 2015). As a central regulator of seed biology, *DOG1* expression is extensively regulated (Tognacca and Botto 2021). Known regulators include 2 long nonprotein coding RNA (lncRNA): one is the *PUPPIES* that activates *DOG1* expression and is transcribed from the *DOG1* promotor (Montez et al. 2023) and a second is *asDOG1* that is transcribed from within the *DOG1* intron 2 in antisense orientation and suppresses *DOG1* expression (Fedak et al. 2016).

Secondary dormancy modulation underlies the dormancy cycling phenomena described for seeds forming soil seed banks (Footitt et al. 2015, 2017). Analysis of histone posttranslational modifications at the *DOG1* gene during dormancy cycling has led to a model where chromatin remodeling at the *DOG1* locus underpins this process (Footitt et al. 2015). Compared to primary dormancy, the mechanisms of secondary dormancy establishment in various plant species including *A. thaliana* are mostly uncharted. Only few regulators have been identified so far and most of them influence both primary and secondary dormancy (Skubacz and Daszkowska-Golec 2017; Buijs 2020). Both types of dormancy are intricately modulated by environmental cues, such as variations in light quality, moisture levels, and transient cold exposure. They are also dependent on internal hormones, namely gibberellic acid (GA) and abscisic acid (ABA, Hauvermale et al. 2015; Sano and Marion-Poll 2021). ABA plays a pivotal role in initiating and sustaining dormancy, while GA acts as the trigger that breaks dormancy and promotes the germination process (Iwasaki et al. 2022).

SWI/SNF (SWItch/Sucrose Non-Fermentable) is a highly conserved chromatin remodeling complex that uses ATP to remodel chromatin. SWI/SNF complexes have been implicated in the control of multiple developmental processes and in orchestrating responses to environmental stimuli in yeast, plants, and animals (Ojolo et al. 2018; Hernández-García et al. 2022; Bieluszewski et al. 2023). In *Arabidopsis*, several homologous subunits of the SWI/SNF complex have been described and many of these subunits are encoded by gene families, including SNF2-type ATPases: SPLAYED (SYD), BRAHMA (BRM), CHR12/MINU1, and CHR23/MINU2 (Guo et al. 2022a; Shang and He 2022). These subunits create the basis for plant SWI/SNF complex taxonomy dividing them into BRM-associated (BAS), SYD-associated (SAS), and MINU-associated (MAS) (Guo et al. 2022b; Fu et al. 2023). The BRM-containing complex (BAS) is probably the best-studied chromatin remodeling complex in plants. BRM ATPase contains multiple protein domains, including a bromodomain that binds acetylated histones and is thought to facilitate the complex recruitment to targeted DNA. In addition to BRM, the BAS complex contains other bromodomain-containing proteins—BRDs (BRD1/2/13) (Jarończyk et al. 2021). Studies of Arabidopsis mutants of BAS complex subunits have shown that the BRM-containing BAS SWI/SNF complex is involved in multiple environmental responses and developmental transitions including seed maturation, embryogenesis, cotyledon separation, leaf development, root stem cell maintenance, floral patterning, or flowering (Yu et al. 2021a, 2021b; Guo et al. 2022b; Shang and He 2022; Bieluszewski et al. 2023). Consistent with these findings, Arabidopsis *brm* knockout mutants (*brm-1*) display severe phenotypes including dwarfism, leaf curling, and sterility (Farrona et al. 2004). Conversely, the *brm-3* mutant, which lacks the bromodomain in the BRM protein, is not sterile and exhibits mild phenotypic abnormalities, including seed coat defects (Hurtado et al. 2006; Farrona et al. 2007). Similar analysis of single and multiple mutants of the *BRD1/2/13* genes revealed their redundant role in regulating vegetative development, flowering, as well as the responses to GA and ABA hormones (Jarończyk et al. 2021; Stachula et al. 2023). Despite the extensive work describing SWI/SNF and BRM's role in seeds development and germination (Han et al. 2012; Ding et al. 2022), the role of the BAS complex in seed biology remains poorly understood.

We and others showed before that apart from promoter regions, chromatin remodelers exhibit extensive binding at the 3′ ends of genes (Brzezinka et al. 2016; Archacki et al. 2016; Jégu et al. 2017). This led us to hypothesize that the BAS-containing SWI/SNF complex may regulate antisense transcription to indirectly control the sense gene expression (Archacki et al. 2016). A reporter–effector study in young Arabidopsis seedlings identified *DOG1* as one of the genes displaying BRM binding at the 3′ end (Archacki et al. 2016). Here, we asked whether the link between BRM and *DOG1* may be important in seeds where the role of DOG1 and its regulation through *asDOG1* is well established (Fedak et al. 2016). Here, we show that BRM is implicated in multiple aspects of seed biology including seed size, seed longevity, and seed dormancy. We demonstrate that some of the affected seed properties, including dormancy, are *DOG1*-dependent and that the BAS complex controls secondary dormancy but not primary seed dormancy through *asDOG1* antisense transcription.

## Results

### BRM-mediated gene expression and metabolite composition in mature *Arabidopsis* seeds

**Figure 1.**
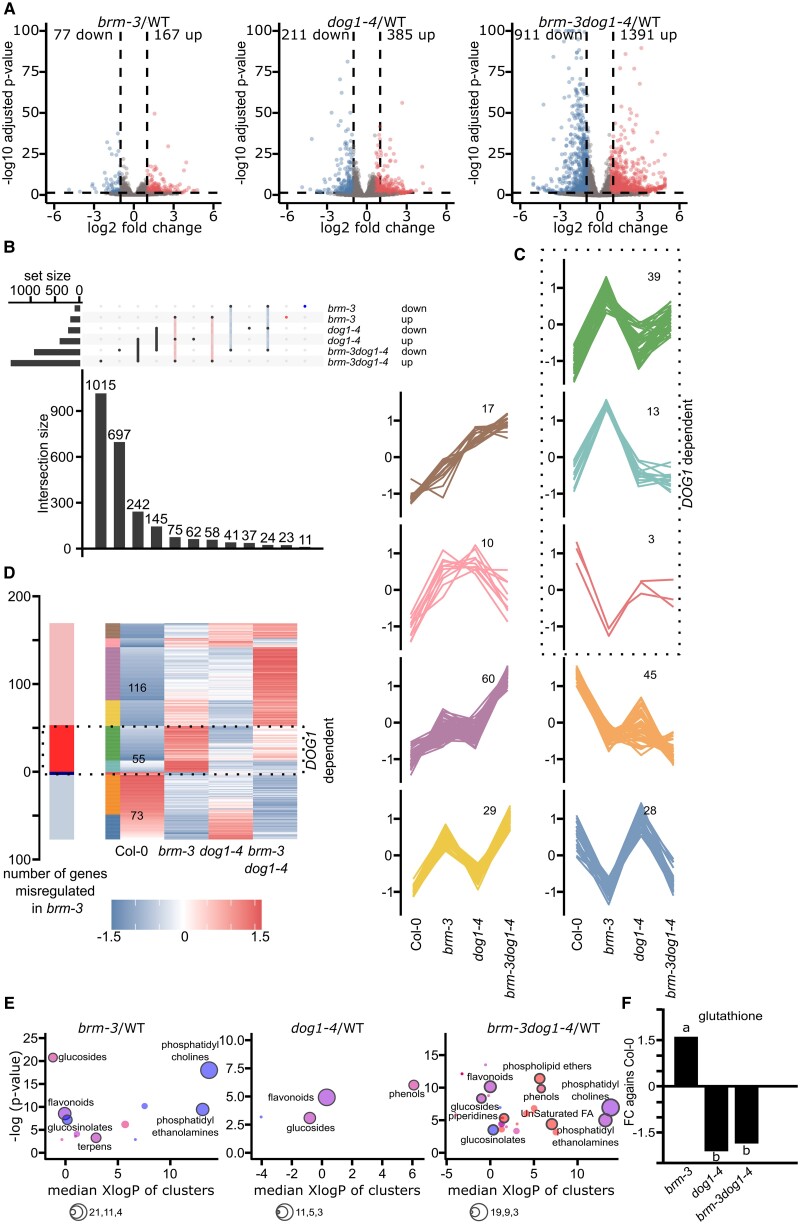
Seeds transcriptome and metabolome of *brm* and *dog1* single and double mutants. **A)** Identification of DEGs in mature, dry *brm-3*, *dog1-4*, and *brm-3dog1-4* seeds compared to Col-0 WT (3′RNA-Seq, differential analysis was performed using DESeq2—genes with FDR < 0.05 and absolute fold change > 1 were considered as differentially expressed). **B)** Analysis of overlap between genes whose expression was affected in analyzed mutants (extended graph version shown in Supplementary Fig. S1C). **C)** Self-clustering of expression profiles for genes differentially expressed in *brm-3*, identifies the *DOG1-*dependent genes among ones affected in *brm* mutant seeds. Number of DEGs is indicated on panels. **D)** Heatmap of genes’ expression for genes misregulated in *brm-3* mutant across mutants used. Genes marked as *DOG1* gene*-*dependent show suppression of the *brm* effect in the double *brm-3dog1-4* mutant. Second column colors correspond to specific color of the cluster. **E)** Chemical enrichment analysis of *brm* and *dog* single and double mutants. Colored circles represent clusters of metabolites from given chemical families (red, increased cluster; blue, decreased cluster; purple, increased and decreased metabolites in a cluster). The number of metabolites as indicated as circle size. **F)** The graph represents the fold change of glutathione levels compared to Col-0 in the seeds of mutants indicated values were ranked into groups as indicated by the respective letter using a Student–Newman–Keuls test, *n* = 4.

To investigate the role of BRM in *Arabidopsis* seed biology in the context of DOG1, we created *brm-3dog1-4* double mutant and compared it with the wild type (WT) and single mutants in downstream analyses. *BRM* knockout allele *brm-1* is sterile, we therefore used the T-DNA insertion line *brm-3* (Tang et al. 2008) and the double mutant *brm-3dog1-4*. 3′RNA-Seq data analysis identified 77, 211, and 911 transcripts with decreased transcript levels in *brm-3*, *dog1-4*, and *brm-3dog1-4*, respectively, and 167, 385, and 1,391 transcripts with increased expression (absolute fold change > 1, FDR < 0.05) (Fig. 1A). As expected, comparison of differentially expressed genes (DEGs) in *brm-3* and *dog1-4* single mutants showed strong overlap with genes misregulated in double *brm-3dog1-4* mutant (Fig. 1B; Supplementary Fig. S1C). Moreover, genes misregulated in *brm-3* and *dog1-4* also showed a substantial overlap (Fig. 1B). Interestingly, *dog1-4*, *brm-3*, and double *brm-3dog1-4* mutants showed a substantial number of genes misregulated in the same directions (up and/or down), suggesting that DOG1 and BRM act synergistically in controlling gene expression in seeds (Fig. 1B). Self-clustering of expression profiles among genes misregulated in *brm-3* identified 9 groups of genes (Fig. 1C). Three groups showed opposite changes in *brm-3* and *dog1-4* and at least partial suppression of the *brm* mutation-caused defects in the double *brm-3dog1-4* mutant (Fig. 1C). Based on this, we considered the BRM effect on those genes as *DOG1* gene-dependent (Fig. 1, C and D).

Transcriptomic analysis showed that both BRM and DOG1 are important players in seed biology and gene expression regulation. Our data revealed that in seeds, BRM and DOG1 control expression of a large number of genes synergistically. There is, however, a substantial subset (55 out of 244) of genes that are regulated by BRM in a DOG1-dependent manner (Fig. 1, C and D).

Gene Ontology (GO) analysis identified 20 and 30 significantly enriched GO terms among genes that were down- and upregulated in the *brm-3* mutant, respectively. Among those, many GO terms represented molecular functions involved in response to oxidative stress, with some related to glutathione metabolism, binding, and transferase activity, which are essential for the control of reactive oxygen species (ROS) (Supplementary Table S1). Single *dog1-4* mutant and *brm-3dog1-4* double mutant showed similar GO term profiles. Those included response to multiple factors, like stimulus or ABA for downregulated genes (Supplementary Fig. S2A), as well as GO terms related to translation—for upregulated genes (Supplementary Table S1).

Next, we analyzed the metabolic profiles of *brm-3, dog1-4*, and *brm-3dog1-4* double mutant mature seeds using a nontargeted comparative metabolomics approach based on high-resolution mass spectrometry. Molecular features detected in each single mutant and in the double mutant *brm-3dog1-4* were compared to Col-0 WT. This identified a total of 410, 112, and 418 differentially abundant molecular features (metabolites) in *brm-3/*WT, *dog1-4/*WT, and *brm-3dog1-4*/WT comparisons, respectively (Wilcoxon rank sum test, fold change ≥ 2; *P*-value < 0.05, Supplementary Fig. S1). Among them, 223 were more abundant and 187 were less abundant in *brm-3* in comparison to Col-0 WT. In *dog1-4* mutant, 30 were more and 82 less abundant when compared to the WT. Finally, in the double mutant, 254 metabolites were more, and 164 were less abundant when compared to Col-0 WT, indicating a significant metabolic remodeling in *brm-3* and *brm-3dog1-4* mutants. Assigned masses allowed us to identify a putative molecular formula for 170 out of 410 differentially expressed metabolites for *brm-3/*WT, 37 out of 112 for *dog1-4/WT*, and 143 out of 418 compounds for double mutant (Supplementary Table S1). To visualize the metabolic changes between mutants and the Col-0 WT, we used a chemical enrichment analysis named ChemRICH (Chemical Similarity Enrichment Analysis for Metabolites) according to Barupal and Fiehn (2017), which provides differentially enriched clusters of metabolites families. Such clusters were identified for each mutant separately (Fig. 1E; Supplementary Table S1). For *brm-3*, we detected 26 differentially abundant metabolic families (*P*-value < 0.05), with a decrease in compounds such as phosphatidylcholine, phosphatidylethanolamine, aldehydes, flavonoids, and glucosinolates and an enrichment of indoles, polyunsaturated alkamides, phenols, unsaturated fatty acids, and amides (Fig. 1E). Interestingly, a 1.6 FC (*P*-value = 0.0012) enrichment in glutathione was found in *brm-3* mutant compared to Col-0 WT (Fig. 1F; Supplementary Table S1). Similarly, in the seeds of the *dog1-4* mutant, metabolomic analysis revealed a decrease in flavonoids and peptides levels and an enrichment in phenols and glucosides (Fig. 1E). Interestingly, in *dog1-4* mutant, we found a −2.08 FC decrease (*P*-value = 0.0059) in glutathione enrichment compared to Col-0 (Fig. 1, E and F). In the double mutant *brm-3dog1-4*, we found a broad variety of metabolic families up- or downregulated compared to Col-0 WT. We noticed an enrichment in phospholipids ethers, phenols, saturated and unsaturated lysophosphatidylcholine, lactones, saturated fatty acids, and indoles; and lower levels of phosphatidylcholine, phosphatydylethanolamine, glucosinolates, flavonoids, and glucosides (Supplementary Table S1). Interestingly, as observed for the single mutants, the double mutant glutathione was downregulated compared to the WT (Fig. 1, E and F; Supplementary Table S1), with a FC of −1.85 (*P*-value = 0.00058). We conclude that similarly to RNA-Seq analysis, metabolomic analysis shows that *dog1* enhances metabolic changes in the *brm-3* background as many changes are only visible or more pronounced in the double *brm-3dog1-4* mutant. In addition to untargeted analysis of metabolites, a targeted analysis of soluble sugars and hormones was performed in the mature seeds of the mutants. This analysis showed that ABA levels were slightly reduced in *brm-3* and *dog1-4* mutant seeds while double mutant seeds showed intermediate levels of ABA, with no significant difference to either Col-0 or single mutants (Supplementary Fig. S3A). The small effect on ABA suggests that phenotypic defects observed in *brm-3* and double mutant are probably not driven through ABA. Similarly, analysis of GA levels in dry seeds showed no significant difference for *brm-3* seeds and higher but not significant levels for *dog1-4* and double mutant seeds (Supplementary Fig. S3B). Given the published role of sugars in seed maturation, we also analyzed levels of sucrose, raffinose, and stachyose (Li et al. 2017; Salvi et al. 2022). Seeds of *brm-3* and *brm-3dog1-4* contained significantly higher levels of raffinose and lower levels of sucrose, resulting in increased RFO/sucrose ratio (Supplementary Fig. S4, A to C). Likewise, stachyose levels were also lower in seeds of the *brm-3* single and *brm-3dog1-4* double mutant (Supplementary Fig. S4D). In contrast, seeds of *dog1-4* mutant did not show changes in sugar levels. These results suggest that BRM is implicated in sugar level control in seeds but independently of DOG1 (Supplementary Fig. S4).

In summary, our metabolomic analysis revealed that *brm-3* and *dog1-4* share some common differentially enriched compounds compared to the WT, while much more diverse families of metabolites are differentially enriched in the double mutant, confirming the synergistic action of BRM and DOG1 in seeds biology (Supplementary Fig. S1; Table S1). Interestingly, our data suggest that the glutathione level is controlled by BRM in a DOG1-dependent manner, as it was increased in *brm-3* mutant, while in *dog1-4* and *brm-3dog1-4* double mutant, glutathione was less abundant compared to Col-0 WT seeds (Fig. 1, F and E; Supplementary Fig. S5).

### BRM regulates seed quality and physiology

**Figure 2.**
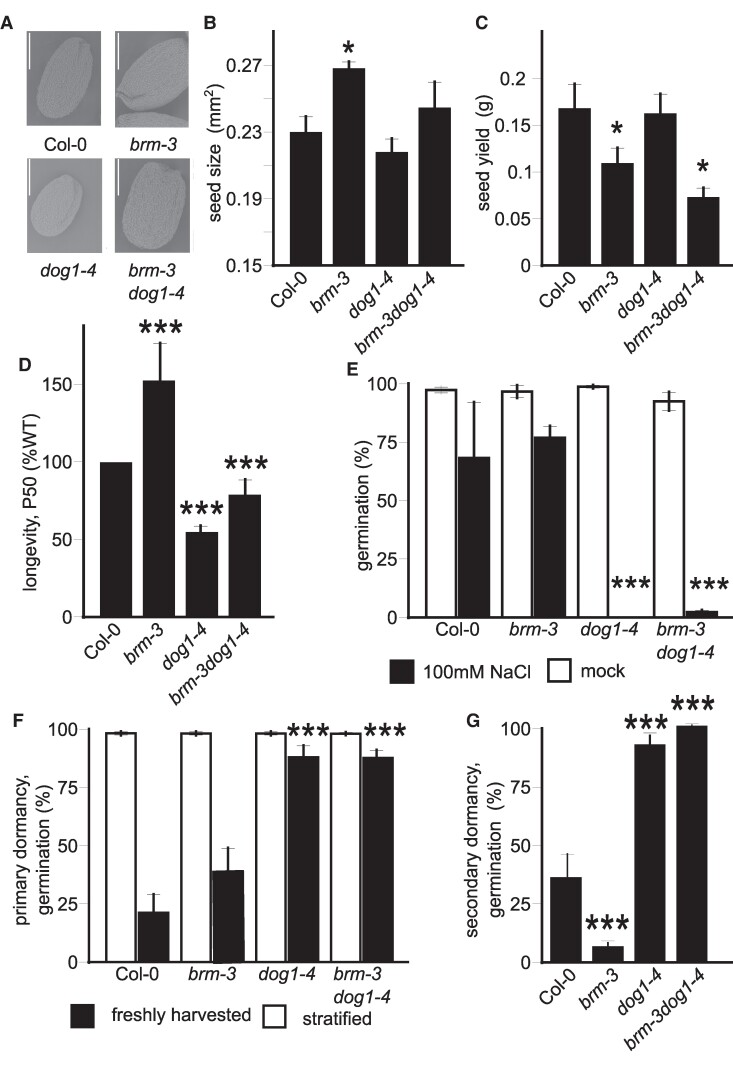
The *brm-3* mutant seeds showed multiple morphological and physiological defects. **A)** Scanning electron visualization of seed from Col-0 WT, *brm-3*, *dog1-4*, and *brm-3dog1-4* mutants. Bar corresponds to 200 nm. **B)** Seeds size analyzed using Boxed robot. **C)** Seed yield analyzed based on total number of seeds produced by mature plants. **D)** Seed longevity analyzed using artificial ageing. **E)** Germination in presence and absence of 100 mm NaCl. **F)** Primary seed dormancy analyzed with freshly harvested seeds. **G)** Secondary dormancy analysis for seed of *brm-3*, *dog1-4*, and *brm-3dog1-4* mutants. Asterisks indicate significant differences compared to Col-0 dry seeds. Statistical analysis applies to all figure panels; *t*-test, *, *P* < 0.05, **, *P* < 0.01 and ***, *P* < 0.0001; *n* = 4, one biological replicate is a mixture of independent 5 plants; error bars represent standard deviation.

To assess the BRM and DOG1 effect on seed quality, we first checked the seed size in single and double mutants (Fig. 2, A and B; Supplementary Fig. S6A). The *brm-3* mutant seeds were 20% bigger compared to the WT and the *dog1-4* mutant, with an average size of 0.27, 0.21, and 0.23 mm^2^ for *brm-3*, *dog1-4*, and the WT, respectively (Fig. 2B; Supplementary Fig. S6A). The *brm-3* mutant seeds were also 25% heavier (*P*-value < 0.00095) compared to the WT. In addition, even though *brm-3dog1-4* seeds were not significantly larger than Col-0 WT seeds, double mutant seeds were significantly heavier than Col-0 seeds (Supplementary Fig. S6D). In addition, the germination rate of *brm3* and *brm-3dog1-4* double mutants in the presence of ABA was similar to Col-0 WT seeds (Supplementary Fig. S6C). As previously reported for *brm-3* (Farrona et al. 2007), *brm-3* and *brm-3dog1-4* mutants had significantly reduced seed yield while *dog1-4* mutant did not show a difference compared to the WT (Fig. 2C). Our data suggest that seed quality measured by seed size, weight but not yield is affected by BRM in a DOG1-dependent manner.

Glutathione is one of the main antioxidants in seeds and its level decreases during seed ageing (Ranganathan and Groot 2023). Our metabolomic analysis showed changes in glutathione levels prompting us to analyze the role of BRM in longevity. Seeds of *brm-3* mutant showed increased longevity compared to the WT (Supplementary Fig. S6B), as seen in the analysis of the time required for 50% loss of viability (P_50_, Fig. 2D). As published by Dekkers et al. (2016), we observed that the P_50_ of the *dog1-4* mutant was lower compared to the WT, demonstrating a role of DOG1 in enhancing longevity. Interestingly, the P_50_ of the double mutant *brm-3dog1-4* was only slightly higher than for the single *dog1-4* mutant. In addition, we performed a germination analysis of 4-year-old naturally aged seeds of the tested mutants. This analysis revealed a germination phenotype similar to that observed in artificially aged seeds: the *brm3* mutant showed significantly higher germination rates compared to Col-0 WT seeds, but this effect was suppressed in the *brm-3dog1-4* double mutant (Supplementary Fig. S6E). This suggests that BRM role in seed longevity is partially DOG1-dependent.

As we found BRM affects seed longevity, we were further interested in its effect on seed's vigor. We analyzed salt sensitivity during germination of after-ripened seeds (Fig. 2E). After-ripened seeds of *brm-3* showed no difference in germination in presence of 100 mm NaCl compared to Col-0. In contrast to what has been observed for freshly harvested stratified seeds, the *dog1-4* mutant showed reduced germination when compared to Col-0 WT (Montez et al. 2023). The *brm-3dog1-4* double mutant behaved like the *dog1-4* single mutant suggesting that BRM is not involved in salt-mediated germination delay (Fig. 2E). Given the role of DOG1 in dormancy, we performed primary and secondary dormancy tests on the mutants. Primary dormancy was tested by germination of freshly harvested seeds and secondary dormancy was analyzed on afterripened seeds pre-treated with high temperature in the darkness (Footitt et al. 2015; Krzyszton et al. 2022). In agreement with published results (Krzyszton et al. 2022), we observed that the *DOG1* gene is required for primary as well as secondary dormancy, as the *dog1-4* mutant showed nearly 100% germination of both freshly harvested seeds and seeds induced into secondary dormancy. In contrast, the *brm-3* mutant displayed a stronger secondary but unaffected primary dormancy when compared to Col-0 WT seeds (Fig. 2, F and G), suggesting a specific function of BRM in secondary dormancy regulation. Likewise for *brm-3*, stronger secondary seed dormancy was also observed in *brm-5*, *3xbrd*, and *swp73a*, other *SWI/SNF* subunit mutants (Supplementary Figs. S7 and S8A).

In summary, we showed that BRM is an important regulator of seed biology required for many aspects of seed development and environmental sensing. Our data reveals a genetic requirement of the functional *DOG1* gene for BRM-mediated control of seed longevity and secondary dormancy. The lack of primary seed dormancy defects and stronger secondary seed dormancy observed in *brm-3* and *brm-5* mutants is surprising, as primary and secondary dormancy levels are usually correlated in Arabidopsis mutants (Buijs 2020; Sajeev et al. 2024).

### BRM binds to the *DOG1* 3′ region and regulates *asDOG1* antisense expression

**Figure 3.**
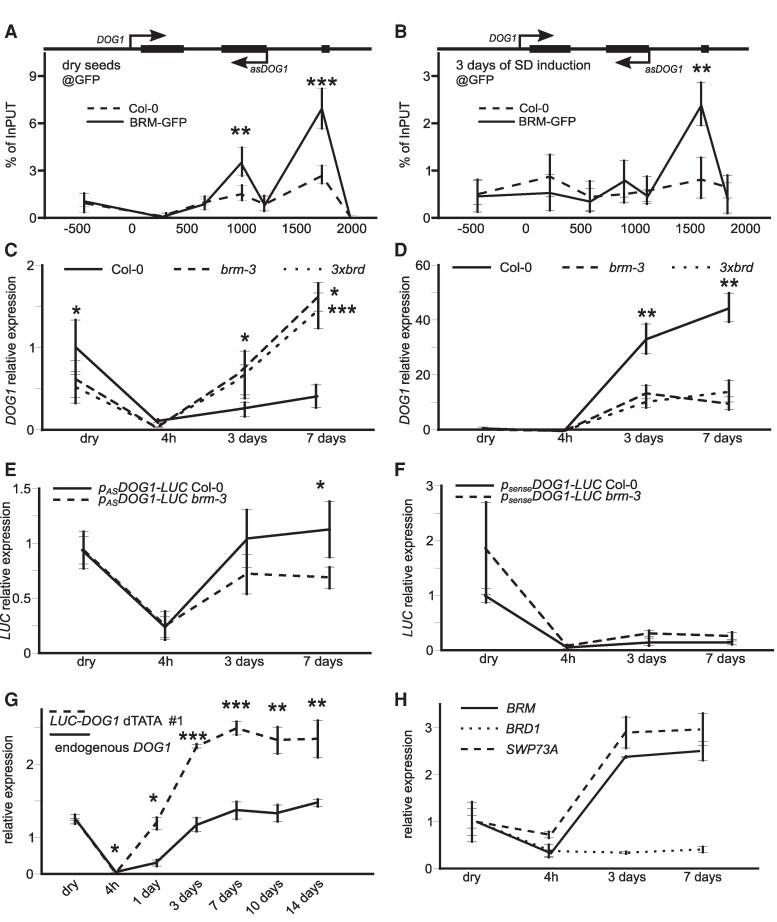
BRM directly regulates *DOG1* antisense transcription to control seed secondary dormancy. **A)** BRM ChIP-qPCR in dry seeds and **B)** seeds subjected to 3 days of secondary dormancy induction. Col-0 and BRM-GFP *brm-1* seeds were analyzed using GFP antibodies. The *x*-axis shows beginning of amplicon relative to TSS, TSS = 0. Percent of input normalized to PP2A gene region. **C)** RT-qPCR analysis of *DOG1* sense and **D)** antisense transcripts in Col-0, *brm-3*, and *3xbrd* mutants during secondary dormancy induction; **E)** RT-qPCR analysis of reporter lines activity during secondary dormancy induction for *p_sense_DOG1-LUC* and **F)**  *p_AS_DOG1-LUC* lines in Col-0 and *brm-3* background. **G)** RT-qPCR for endogenous and *LUC-DOG1*-*deltaTATA* line activity during secondary dormancy induction shows that inactivation of *asDOG1* results in stronger induction of *DOG1* during secondary dormancy induction. **H)** BRM, SWP73A, and BRD1 genes expression analysis during SD induction in *Arabidopsis* seeds. RT-qPCR analysis in **C** to **H)** is normalized using *UBC21* gene. Statistical analysis applies to all figure panels, *t*-test, *, *P* < 0.05, **, *P* < 0.01 and ***, *P* < 0.0001; *n* = 4, one biological replicate is a mixture of independent 5 plants; error bars represent standard deviation.

Our genetic analyses suggested that BRM-mediated regulation of secondary dormancy requires the *DOG1* gene. Previously, it has been shown that the *DOG1* gene 3′ end is bound by BRM and BRD in seedlings (Archacki et al. 2016; Yu et al. 2020, 2021a, 2021b; Supplementary Fig. S9, A and B). To confirm BRM binding at the *DOG1* locus in seeds, we performed ChIP-qPCR experiment using a transgenic line expressing BRM-GFP under a native promoter in the background of *brm-1* null mutant (Jarończyk et al. 2021). We observed BRM binding mostly within exon 2 and exon 3 of the *DOG1* gene, matching the location of previously described by us antisense lncRNA *DOG1* promoter (Fig. 3A). This suggests that BRM could control the *DOG1* gene expression in seeds via as*DOG1* (Fedak et al. 2016).

In nondormant dry seeds, BRM was bound at exon 2 and exon 3 regions (*asDOG1* promoter), similar to genome-wide ChIP data from Arabidopsis seedlings (Fig. 3B; Supplementary Fig. S9). During SD induction, BRM was predominantly bound at exon 3 (Fig. 3B). This suggests that secondary dormancy induction may lead to changes in the way BRM controls *DOG1* sense and antisense expression. This is in agreement with our phenotypic analysis (Fig. 2G; Supplementary Fig. S7) that demonstrated that *brm-3* seeds show an enhanced propensity to enter secondary dormancy while seeds collected from *brm-3dog1-4* double mutant are unable to enter dormancy.

Next, we performed RT-qPCR analysis of *DOG1* gene expression in the Col-0 WT and selected mutants during secondary dormancy induction. Upon imbibition at 4 h, we observed a strong *DOG1* mRNA reduction followed by a gradual rebuild of *DOG1* mRNA levels during secondary dormancy induction (Fig. 3C). This is in agreement with previously published by us and others’ results (Buijs 2020; Krzyszton et al. 2022; Sajeev et al. 2024). Interestingly, we observed a much stronger increase in *DOG1* mRNA levels in *brm-3* mutant (Fig. 3C). To test if the observed BRM role in *DOG1* expression regulation during secondary dormancy induction requires activity of the whole BAS complex, we used a single *swp73a* and a triple *BRD1/2/13* (*3xbrd*) mutant that both are components of BAS SWI/SNF complex (Guo et al. 2022a, 2022b; Fu et al. 2023). We observed hyper activation of *DOG1* expression in *3xbrd* and *swp73a* mutant seeds (Fig. 3C; Supplementary Fig. S8B) that was very similar to the one observed in *brm-3*. We also note a stronger dormancy phenotype during secondary dormancy induction for *3xbrd* and *swp73a* mutants (Supplementary Figs. S7 and S8A). The observed upregulation of *DOG1* expression in the *brm-3*, *swp73a*, and *3xbrd* mutants suggests a previously unrecognized role for BRM and the SWI/SNF BAS complex in the suppression of *DOG1* expression during secondary dormancy establishment. Gene expression analysis showed an increase in the mRNA levels of the *BRM* and *SWP73A* genes but not for *BRD1*, suggesting that the main SWI/SNF subunits are co-induced with *DOG1* gene during secondary dormancy establishment. However, BRM expression was not affected in *dog1-4* mutant compared to Col-0 WT seeds during induction (Supplementary Fig. S10A). Thus, our genetic and RT-qPCR analyses suggested that BRM function upstream of the DOG1 gene.

In parallel to sense transcript expression during secondary dormancy induction, we analyzed *asDOG1* transcript levels. Similarly, to sense transcript expression, we observed a gradual accumulation of antisense transcript during dormancy induction (Fig. 3D). All *brm-3*, *swp73a*, and 3x*brd* mutants showed a clear reduction in the levels of *asDOG1* expression when compared to the Col-0 seeds at later stages of dormancy induction (Fig. 3D; Supplementary Fig. S9C). Together with BRM binding at *DOG1* 3′ end this suggests that BRM directly regulates *asDOG1* transcription. To test this possibility, we used the p*_AS_DOG1*promoter-driven IRES-LUC reporter line and crossed it with the *brm-3* mutant. RT-qPCR using LUC primers showed significant downregulation of *LUC* transcript in *brm-3*, suggesting that BRM directly regulates *asDOG1* promoter activity (Fig. 3F).

### BRM regulation of *DOG1* gene expression requires *asDOG1*

Observed by us direct regulation of *DOG1* antisense expression by BRM and the lack of BRM binding to canonical *DOG1* promoter suggested a model where BRM regulates *DOG1* sense expression through *DOG1* antisense. To test this model, we first asked if BRM can regulate *DOG1* expression in the absence of as*DOG1*. We used a previously published (Fedak et al. 2016) transgenic truncated *DOG1* gene (*_pDOG1_shDOG1::LUC*) with an antisense promoter deleted and crossed it to a *brm-3* mutant (Fig. 3E). Importantly, no significant differences were observed between Col-0 and *brm-3*, suggesting that BRM is unable to regulate the *DOG1* gene expression when the *DOG1* 3′ region is removed. Surprisingly, *p_DOG1_shDOG1::LUC* did not show the induction of expression that we observed for endogenous sense mRNA.

Therefore, the inability of BRM to regulate the *DOG1* gene with antisense deleted (*p_sense_DOG1-LUC*), together with the fact that in *brm-3* mutant *p_AS_DOG1* activity is suppressed, suggest that BRM acts through antisense. The fact that *p_sense_DOG1-LUC* transgene did not recapitulate endogenous *DOG1* sense expression inductions suggests that some of the elements required for *DOG1* induction upon secondary dormancy induction are located in regions deleted in the construct.

To test this possibility, we took advantage of a set of TATA mutations shown by us previously to greatly reduce *asDOG1* transcript expression (Yatusevich et al. 2017). We engineered these mutations in an antisense promotor of the *DOG1* genomic reporter construct creating *LUC-DOG1-deltaTATA* transgenic lines. RT-qPCR analysis using primers that amplify only transgene-originating *DOG1* mRNA showed that removal of antisense transcription indeed resulted in strong upregulation of *DOG1* sense transcript at early stages of dormancy induction (Fig. 3G; Supplementary Fig. S11, A and B). Together the transgenes analysis shows that BRM requires *asDOG1* to control *DOG1* expression during secondary dormancy induction and that *asDOG1* acts as negative regulator of *DOG1* expression during secondary dormancy establishment. Similar to *brm-3* and *brm-5* mutants, transgenic *LUC-DOG1* lines carrying dTATA mutations showed stronger secondary seed dormancy compared to the WT *LUC-DOG1* lines, confirming *asDOG1* acts as a negative regulator of *DOG1* expression during secondary dormancy (Supplementary Fig. S11C).

**Figure 4.**
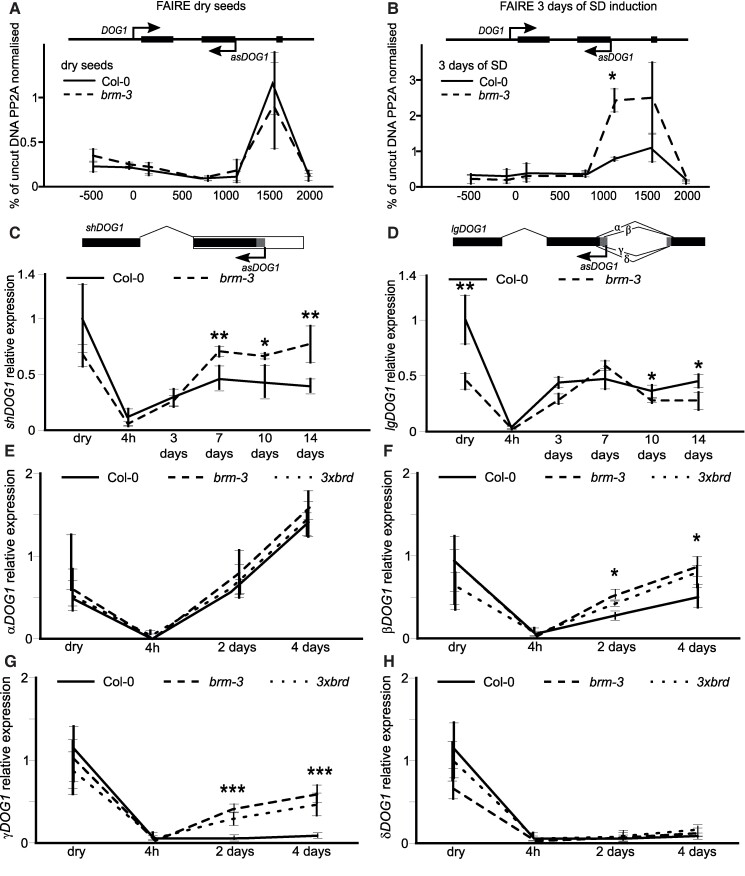
The *brm3* mutant shows enhanced chromatin accessibility at *DOG1* 3′ end during secondary dormancy induction. **A)** FAIRE in Col-0 and *brm-3* seeds on the 3rd day and **B)** 5th day of secondary dormancy induction. Chromatin accessibility at *DOG1* shown as % recovery to noncrosslinked samples (UNFAIRE) and relative to PP2A. The *x*-axis shows beginning of amplicon relative to TSS, TSS = 0. **C** to **F)** RT-qPCR analysis of *α-, β-, γ-*, and *δ-DOG1* mRNA splicing forms during secondary seed dormancy induction. Transcript level of short **G)** and long **H)** polyadenylated *DOG1* mRNA forms The *x*-axis shows time/days of secondary dormancy induction. Statistical analysis applies to all figure panels; *t*-test, *, *P* < 0.05, **, *P* < 0.01 and ***, *P* < 0.0001; *n* = 4, one biological replicate is a mixture of independent 5 plants; error bars represent standard deviation.

BRM is part of the SWI/SNF complex and our data show that both BRM and another SWI/SNF complex subunits—BRDs are implicated in seed secondary dormancy control through *asDOG1* antisense transcript promoter regulation. SWI/SNF is a chromatin remodeling complex that utilizes ATP to remodel chromatin at target loci (Mashtalir et al. 2018). To test if BRM-mediated regulation of *DOG1* sense expression through *asDOG1* is accompanied by DNA accessibility changes, we performed FAIRE (Formaldehyde-Assisted Isolation of Regulatory Elements) during secondary dormancy induction (Omidbakhshfard et al. 2014). On the third day of secondary dormancy induction, *brm-3* showed a marked increase in DNA accessibility using FAIRE at the end of exon 2 compared to dry seeds (Fig. 4, A and B). This increase was even more pronounced in the last intron and exon 3 of the *DOG1* region on the 5th day of induction, centering around the *DOG1* antisense promoter region (Supplementary Fig. S8D). This region colocalized with the BRM binding site identified in ChIP experiments (Figs. 3, A and B; 4, A and B; Supplementary Fig. S8, C and D). In agreement with BRM involvement in *DOG1* regulation during secondary dormancy, the FAIRE experiment in dry seeds failed to detect a localized increase at *DOG1* 3′ end in *brm-3* compared to Col-0 WT dry seeds (Fig. 4A). Thus, this suggests that during secondary dormancy induction, BRM is directly bound at *DOG1* 3′ end and locally remodels chromatin presumably to regulate *asDOG1* expression. This is consistent with the *asDOG1* requirement for BRM's ability to suppress *DOG1* gene expression during secondary dormancy induction.

### BRM affects *DOG1* splicing and polyadenylation

To gain a deeper understanding of *DOG1* regulation by BRM, we analyzed the misregulation of *DOG1* splicing and polyadenylation in *brm-3* seeds during the induction of secondary dormancy. Our RT-qPCR analysis examined early time points (2- and 4-days postinduction) for splicing and up to 14 days postinduction for polyadenylation. We detected increased levels of the short proximally polyadenylated *DOG1* mRNA (*shDOG1*), but not the long form (*lgDOG1*), during secondary dormancy induction in *brm-3* (Fig. 4, C and D). We also found a significant increase in *β* (beta) and *γ* (gamma) *DOG1* mRNA splicing forms in *brm3* mutants compared to Col-0 seeds (Fig. 4, E to H). Increased *β*, *γ*, and *shDOG1* were not only observed in *brm-3* but also in *3xbrd* mutant, suggesting that they are a result of BRM activity linked to the SWI/SNF complex. The increase in *β*, *γ*, and *shDOG1* mRNA is consistent with the observed stronger *brm-3* mutant secondary seed dormancy phenotype as *shDOG1* has been reported to be the predominant *DOG1* isoform that can complement the *DOG1* mutant phenotype and *β* and *γ* mRNA isoforms lead to production of the same protein as encoded by *shDOG1*. Interestingly, in the *ntr1* mutant, known as a spliceosome disassembly factor (Dolata et al. 2015), we observed a significant reduction in *αDOG1* mRNA splicing forms, while other *β*, *γ*, and δ splicing forms showed similar kinetics to one observed in Col-0 WT (Supplementary Fig. S15). The differential effect of *brm-3* and *ntr1* on *DOG1* splicing suggests that BRM does not control *DOG1* splicing through NTR1.

## Discussion

### BRM-containing SWI/SNF complex controls seed physiological quality

We show that *brm-3* mutants exhibit multiple seed-related phenotypes, including enlarged seed size, reduced seed yield, increased seed longevity, and enhanced secondary dormancy induction but no change in primary dormancy or germination in the presence of salt (Fig. 2; Supplementary Fig. S14). Given the central role of the *DOG1* gene in seed biology as well as BRM binding to *DOG1* locus, we tested the interplay of *BRM* and *DOG1* genes in seeds. We found that some of the *brm-3* mutant phenotypes are genetically dependent on *DOG1* gene as a double mutant of *brm-3dog1-4* shows a reversal of the longevity and secondary dormancy phenotypes (Fig. 2). Transcriptomic analysis in dry seeds showed that around 20% of genes misregulated in *brm-3* are *DOG1* gene-dependent as *dog1-4* mutation can partially or fully suppress the *brm* mutation effect on their expression in the *brm-3dog1-4* double mutant (Fig. 1D). In addition, we observed a pronounced misregulation of gene expression in the double *brm-3dog1-4* double mutant (Fig. 1A) which suggests a synergistic function in the case of the majority of affected genes.

### Longevity and metabolite accumulation

GO terms analysis among differentially regulated genes suggested changes in genes involved in the biosynthesis of metabolites known to be important in seed biology (Supplementary Table S1) that were mostly consistent with changes observed in seed metabolome analysis (Supplementary Fig. S1, A and B). RNA-Seq data showed that genes related to glutathione metabolism were enriched among genes downregulated in *brm-3dog1-4* double and *dog1-4* single mutant and upregulated in single *brm-3* mutant (Supplementary Figs. S2 and S5). This is consistent with our metabolomic analysis that showed an increase in *brm-3* and lower levels of glutathione in *dog1-4* and *brm-3dog1-4* mutants (Fig. 1F; Supplementary Fig. S5). Given the published link between glutathione level, dormancy, and longevity in Arabidopsis (Cairns et al. 2006; Nguyen et al. 2015; Koramutla et al. 2021), we note that the observed changes in glutathione level are probably responsible for the observed *DOG1* gene-dependent partial increase in longevity of *brm-3* mutant. This observation is corroborated by the previously suggested positive role of *DOG1* in longevity based on analysis of natural variation and *DOG1* NIL line analysis (Nguyen et al. 2012).

Changes in soluble sugar contents have been suggested to be involved in germination and longevity regulation (He et al. 2016). In legume species, a correlation between lower seed storability and a lower ratio between RFO and sucrose has been reported (Pereira Lima et al. 2017). While in Arabidopsis, this correlation remains unclear, as an increase in the RFO/sucrose ratio was not found to be correlated with seed vigor (Bentsink et al. 2000; Li et al. 2017). We found a significant decrease in the RFO/sucrose ratio in both single *dog1-4*, *brm-3*, and double mutants (Supplementary Fig. S4B). However, there is no significant difference in this ratio between *dog1-4* and *brm-3* mutants, despite the difference in seed longevity between them. Thus, our data suggest that in Arabidopsis, there might be no direct link between RFO and seed longevity. However, it is interesting to further investigate galactinol contents as it has been linked to seed biology and has not been measured by us (De Souza Vidigal et al. 2016).

In addition, in dry *brm-3* mutant seeds, we observed significant alterations in various classes of tryptophan-derived metabolites, including auxin, camalexin, and indole-glucosinolates. The deregulation of genes involved in the tryptophan-derived metabolite pathways (such as *MYB34*, *MYB51*, MYB122, *NITs*, *ARFs*, and *CYP79B3*) in the *brm* mutant may contribute to the observed auxin-related phenotypes and reduced seed yield of *brm-3* (Supplementary Fig. S2B). While camalexin and indole-glucosinolates are recognized as plant-defensive secondary compounds against pathogens and herbivores (Stotz et al. 2011; Nguyen et al. 2020), their specific biological roles in seeds dormancy remain to be fully elucidated.

### The role of BRM in glutathione accumulation in seed is *DOG1*-dependent

Glutathione is an important player in redox signaling and is involved in protection against excessive oxidation in multiple plant tissues (Mhamdi et al. 2010). Accumulation of oxidative damage during dry seed storage is probably the most important factor behind deterioration of seed quality and eventually loss of viability determining seed longevity (Kumar et al. 2015).Our transcriptomic analysis showed that genes related to glutathione metabolism were misregulated in *brm-*3 mutant in the opposite direction to changes observed in *dog1-4* mutant, including *GPX1* and *GPX6* that are responsible for glutathione biosynthesis. We also observed multiple other misregulated genes in different pathways related to glutathione synthesis, degradation, and recycling. Genes coding for GLUTATHIONE S-TRANSFERASEs: GSTU9, GSTU10, GSTU11, GSTU12, and GSTU19 were significantly upregulated (FDR < 0.05, FC > 2) in mature seeds of *brm-3* mutant compared to Col-0 WT seeds (Fig. 1F; Supplementary Figs. S2 and S5). No consistent change of the transcript levels of these genes were observed in *dog1-4* seeds.

In agreement with the observed deregulation of glutathione-related transcripts, we observed a higher level of glutathione in the *brm-3* mutant, and depletion in *dog1-4* and double mutant compared to Col-0. Those results are concordant with the reduced longevity in both *dog1-4* and double mutant, and the increased longevity of the *brm-3* mutant.

### ABA and GA hormonal signaling in *brm-3* mutant seeds during SD

Our analysis of hormone levels in dry seeds revealed relatively minor differences in the single mutants *brm-3* and *dog1-4* regarding GA and auxin content (Supplementary Fig. S3, B and C). Interestingly, we observed a significantly elevated auxin level in the *brm-3 dog1-4* double mutant (Supplementary Fig. S2C), correlating with increased expression of auxin pathway genes (Supplementary Fig. S7B). A slight reduction in ABA levels was detected in *dog1-4* and slightly in the double mutant (Supplementary Fig. S3A). Members of the SWITCH2 (SWI2)/SNF2 chromatin remodeling complexes play a role in seed germination under ABA treatment. BRAHMA (BRM) directly suppresses the expression of ABI5 and, consequently, the *brm-3* mutant exhibits increased ABA sensitivity during seed germination (Han et al. 2012). The role of ABA and its signaling pathway in seed biology has been extensively studied, including its role in secondary seed dormancy establishment (Auge et al. 2015; Ibarra et al. 2016).

In agreement with published results, we have previously reported that quadruple *nced2569* mutant failed to enter into secondary dormancy (Lefebvre et al. 2006; Krzyszton et al. 2022). Here, we show that *nced2569* shows no defect in *DOG1* expression during secondary dormancy induction when compared to Col-0 seeds (Supplementary Fig. S10B). Also, analysis of the expression of genes related to ABA biosynthesis and catabolism (*NCED4/5* and *CYP707A2*) showed only minor fluctuations during secondary dormancy induction in Col-0 seeds and no major differences when compared to *brm-3* or *dog1-4* mutants (Supplementary Figs. S12A and S13A). In contrast, we observed a strong induction of *RGL1*, *RGL2*, and *GAI* genes—known negative regulators of the GA pathway, during secondary dormancy induction. Interestingly, *RGL1* and *RGL2* but not *GAI* showed strong upregulation in *brm-3* mutant when compared to the WT seeds during dormancy induction (Supplementary Fig. S12B). In contrast, in *dog1-4* mutant, only *RGL1* and *GAI* genes were induced (Supplementary Fig. S13B). While we did not analyze the levels of gibberellins during secondary dormancy induction, this may suggest a role of GA catabolism genes rather than ABA in enhanced secondary dormancy induction in *brm-3* (Ibarra et al. 2016; Chang et al. 2018) and required functional *DOG1*.

### BRM controls secondary seed dormancy through *DOG1* antisense

In agreement with the genetic interplay between *BRM* and *DOG1* genes, we detected direct BRM binding to exon 2 and exon 3 of the *DOG1* gene in dry seeds. Interestingly, during secondary dormancy induction, BRM binding increased toward the 3′ end of the *DOG1* locus (Fig. 3, A and B). Together with the observed deregulation of the *DOG1* gene expression and changes in chromatin accessibility, this suggests that BRM controls secondary dormancy directly through *DOG1*. Primary and secondary dormancy are intrinsically linked and multiple factors including DOG1, AFP2, ABI5, and ABI3 have been shown to affect both primary and secondary dormancy (Han et al. 2012; Ibarra et al. 2016; Chang et al. 2018). Here, we show that BRM is specifically implicated in secondary but not primary dormancy control. To the best of our knowledge, this is the first example of where a factor is required only for secondary but not primary seed dormancy.

DOG1 is a known positive regulator of dormancy (Bentsink et al. 2006). Here, we show that during secondary dormancy induction, sense *DOG1* transcript is induced (Fig. 3C). This is in agreement with published by us and others requirement of functional *DOG1* gene for secondary dormancy establishment (Ibarra et al. 2016; Krzyszton et al. 2022; Sajeev et al. 2024). Our data show that in *brm-3* seeds, *DOG1* transcript is upregulated while *asDOG1* is downregulated during secondary dormancy induction, when compared to Col-0 (Fig. 3, C and D). We also observe BRM binding to *DOG1* 3′ end region, and that BRM regulates *asDOG1* but not *DOG1* sense promoter activity during secondary dormancy establishment (Fig. 3). Supporting a direct role of BRM in control of *asDOG1* we observed increased DNA accessibility at *asDOG1* promoter region of the *DOG1* locus in *brm-3* mutant during secondary dormancy establishment (Fig. 4A). Interestingly, BRM binds to the 3′ end region of selected DEGs involved in ABA and GA pathways (such as *RGL3*, *NCED4*, and *CYP707A1*) (Supplementary Table S2).

**Figure 5.**
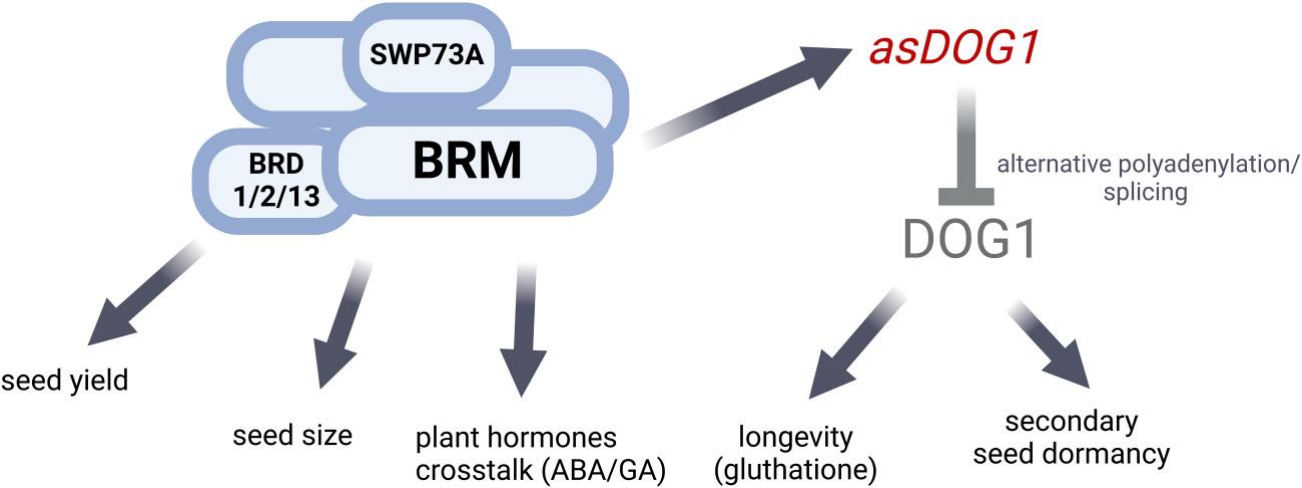
Model of the BRAHMA-associated SWI/SNF complex control of *Arabidopsis* seeds quality and physiology. The BRAHMA-associated SWI/SNF complex controls seed yield, seed size, and plant hormonal crosstalk to a large extend independently of DOG1. The BRAHMA controls longevity and secondary dormancy by controlling *DOG1* expression through *DOG1* antisense (in red color; dark gray arrows). The BRAHMA also either directly or through *DOG1* antisense negatively controls *DOG1* gene expression (gray arrow) by affecting its alternative splicing and alternative polyadenylation.

Previous research has shown that *asDOG1* is a negative regulator of *DOG1* expression, as its deletion results in high *DOG1* expression (Fedak et al. 2016; Yatusevich et al. 2017). BRM appears to positively control antisense, thus also negatively regulating *DOG1* gene expression (Figs. 4 and 5). The mechanism of *DOG1* silencing by its antisense, aka *1GOD*, is not yet fully understood. We hypothesize that during secondary dormancy establishment, BRM indirectly limits *DOG1* induction by enhancing *asDOG1* expression (Fig. 3D). In agreement, we show that mutation of TATA boxes located in the antisense promoter region resulted in much stronger *DOG1* upregulation compared to not mutated *DOG1* transgene and, in agreement, enhanced secondary dormancy phenotype in seeds. Our model suggests that observed by us in Col-0 upregulation of *asDOG1* during secondary dormancy induction serves to limit *DOG1* induction attenuating dormancy strength. In contrast to BRM function in secondary dormancy, both RNA-Seq and RT-qPCR showed no major differences in *DOG1* sense and antisense transcripts levels between Col-0 and *brm-3* mutant in dry seeds. This agrees with the observed lack of primary dormancy defects in *brm-3* as well as in *3xbrd* and *swp73a* mutants (Fig. 2F; Supplementary Fig. S14).

Notably, the introduction of triple *3xbrd* mutation into the *brm-1* knockout mutant background did not enhance the *brm-1* phenotype, confirming the conclusion that BRD subunits operate within the same complex as BRM (Stachula et al. 2023). Also, here we show that *3xbrd* and *swp73a* display similar *DOG1* expression changes to *brm-3* mutant consistent with BRM operating as part of BAS SWI/SNF complex in controlling *DOG1* expression (Fig. 3; Supplementary Fig. S9).

Thus, we propose a model where BRM-containing the SWI/SNF complex binds to the *DOG1* 3′ end region and in response to secondary dormancy-inducing conditions remodels nucleosomes which activate *asDOG1* antisense promoter (Fig. 5). This leads to *asDOG1* transcript expression that limits the activation of *DOG1* and subsequently to a strong dormancy establishment. Surprisingly, analysis of *p_sense_DOG1-LUC* transgene that lacks a function *DOG1* antisense transcript in seeds showed that *DOG1* 5′ region is insufficient to support *DOG1* expression upregulation in response to secondary dormancy induction. This suggests that in addition to *DOG1* antisense that limits the full activation of *DOG1* expression, the *DOG1* 3′ end region contains unknown positive regulators responsible for secondary dormancy-mediated *DOG1* expression induction.

Analysis of *DOG1* splicing and polyadenylation during secondary seed dormancy induction in Col-0 WT, *brm3* showed increased levels of *β* and *γ* alternatively spliced mRNA as well as increased levels of *shDOG1* resulting from selection of proximal termination site (Fig. 4, F and G). Whereas in the *ntr1* mutant, we observed predominantly changes in *α* and not significantly in *β DOG1* mRNA splicing forms compared to Col-0 WT seeds (Supplementary Fig. S15). Given that our work implicated NTR1 in splicing control through Pol II speed on *DOG1*, we speculate that defects observed in *brm-3* are unlikely to results from NTR1-dependent splicing defects or direct Pol II speed regulation. One possibility is that changes in *DOG1* splicing and termination site selection observed in *brm-3* and *3xbrd* result from defects in antisense expression in these mutants. The proximity of *DOG1* alternative splice sites—proximal termination site and antisense promoter make deletional confirmation of this hypothesis difficult, if possible. We, however, showed that BRM regulates the *DOG1* antisense promoter in seeds in the absence of sense promoter driving *DOG1* alternative splicing or proximal termination site selection.

In summary, our work explores the functions of the BRM-containing SWI/SNF complex in seed biology. We observe that BRM is required for multiple aspects of seed physiology as underpinned by metabolomic and transcriptomic analysis. We show that BRM controls some of the seed-related phenotypes including longevity and secondary dormancy regulation through the *DOG1* gene. Our analysis demonstrates that in response to environmental signals triggering secondary dormancy induction BRM-containing SWI/SNF complex controls *DOG1* expression through *DOG1* antisense.

## Materials and methods

### Plant material and growth conditions

*A. thaliana* plants were grown in pots with mixed coconut and normal soil in a greenhouse with a long-day photoperiod (16 h light/8 h dark) at 22 °C/18 °C. After harvest, seeds were stored in paper bags at room temperature (RT). Ecotype Col-0 plants were used as a WT. The *brm-3* (SALK_088462) and *dog1-4* (SM_3_20886) mutants were obtained from the Nottingham Arabidopsis Stock Centre (NASC) and are described in Farrona et al. (2007) and Fedak et al. (2016). Transgenic reporter lines: genomic *LUC-DOG1dTATA*, *asDOG1dTATA-LUC*, *senseDOG1-LUC*, *asDOG1-LUC*, and g*enomicLUC-DOG1* were generated and characterized previously (Fedak et al. 2016; Yatusevich et al. 2017). The *brm-5*, *swp73a* (SM_3_30546), *3xbrd, ntr-1, 4xnced*, and *brm-1*/*BRM-GFP* lines were described previously (Tang et al. 2008; Dolata et al. 2015; Sacharowski et al. 2015; Jarończyk et al. 2021; Krzyszton et al. 2022). The double mutants such as *brm-3dog1-4*; *brm-3p_sense_DOG1-LUC*; *brm-3p_AS_DOG1-LUC*; *brm-3genomic LUC-DOG1dTATA*; and *brm-3p_AS_DOG1dTATA-LUC* were generated by crossings, and homozygous plants were identified using *brm-3* T-DNA insertion primers described in Jarończyk et al. (2021).

For salt stress, sterilized after-ripened seeds were sown on agar plates supplemented with 100 mm NaCl. After sowing, plates were taped, wrapped with silver foil, and kept for stratification for 3 days at 4 °C. Then, plates were unwrapped from the silver foil and transferred to the growth chamber under long-day conditions (16 h light/8 h dark) to check the germination rate (Montez et al. 2023).

### Seed longevity measurement

To perform artificial ageing, mature postharvested *Arabidopsis* seeds were stored in the darkness at 35 °C in hermetically closed containers with saturated NaCl (75% of relative humidity). Fifty seeds per each biological replica were imbibed on blue paper (Anchor) plates after different storage times (0, 7, 14, 21, 28, 35, 42, 49, and 56 days) and placed in a phytotron at 22 °C/long-day photoperiod. The final germination percentage was counted after 10 days. P_50_ was determined as the time after which seeds lost 50% of their germination capacity (Zinsmeister et al. 2020).

### Seed primary dormancy assay

Freshly harvested seeds were sown on plates with water-soaked blue paper (Anchor) and sealed with tape. Plates with the seeds were put in the growth chamber under long-day conditions (16 h light/8 h dark) at 22 °C/18 °C. The germination rate was scored every day until 100% germination was observed. Control plates were initially stratified for 3 days at 4 °C to break seed dormancy and to ensure that the seeds were not dead.

### Seed secondary dormancy assay

Seeds stored for at least 3 months that showed full loss of primary dormancy were used for secondary dormancy induction. Seeds were sown on water-soaked blue paper plates, sealed and kept in the dark. Plates were incubated at 30 °C for 4 h, 1, 3, 5, 7, 10, or 14 days. After high incubation, the plates were transferred to the growth chamber at 22 °C under long-day conditions. Seed germination was assayed after 4 and 7 days. The control plates were placed in a phytotron immediately after sowing the seeds, without a dormancy induction.

### RNA extraction, cDNA synthesis, and RT-qPCR analysis

RNA extraction from seeds was performed using the phenol–chloroform protocol. The frozen seeds were ground to a powder using an electric drill and then mixed with 600 *µ*l of RNA extraction buffer (100 mm Tris pH 8.5, 5 mm EDTA pH 8.0, 100 mm NaCl, 0.5% SDS, 1% β-mercaptoethanol). Afterwards, samples were centrifuged for 5 min at 14,000 × *g* at 4 °C. The supernatant was transferred to new tubes and 250 *µ*l of chloroform was added and samples were shaken at RT for 15 min. Then 250 *µ*l of phenol was added and samples were shaken for a further 15 min and centrifuged for 10 min at 14,000 × *g* at 4 °C. Then, 550 *µ*l of the aqueous layer was transferred to new tubes and mixed with 550 *µ*l of phenol–chloroform–isoamyl alcohol 25:24:1. Samples were shaken for 10 min at RT and centrifuged for 10 min at 14,000 × *g* at 4 °C. Then, 500 *µ*l of supernatant after transferring to new tubes was mixed with 50 *µ*l of 3 m sodium acetate and 400 *µ*l of pure ice-cold isopropanol and incubated for 15 min at RT. After the incubation, samples were centrifuged for 30 min at 14,000 × *g* at 4 °C. Finally, the RNA pellet was washed in 1 ml of 80% ethanol, dried and resuspended in Milli-Q water. DNase treatment of RNA samples was performed using a TURBO DNA-free Kit (Thermo Fisher Scientific), according to the manufacturer's protocol. DNase treatment effectiveness was checked by PCR with pp2A primers. Reverse transcription of RNA was performed using a RevertAid First Strand cDNA Synthesis (Thermo Fisher Scientific) or RevM First Strand cDNA Synthesis (KleverLab) kits according to the manufacturer's protocol. Two types of cDNA synthesis were performed: using 1,000 ng of RNA and oligo(dT) primers for *DOG1* analysis and gene-specific synthesis for *asDOG1* analysis using 2,500 ng of RNA and primers with overhangs as described (Fedak et al. 2016). qPCR was performed with SYBR Green mix and specific primers for PCR amplification and with using LightCycler 480 real-time system (Roche). The sequences of all primers are published previously (Cyrek et al. 2016; Fedak et al. 2016) and provided in Supplementary Table S2. RT-qPCR results were normalized to the expression level of the *UBC21* (AT5G25760) gene.

### RNA-Sequencing and data analysis

3′RNA-Seq and data analysis were performed as described previously using 500 ng of total RNA as starting material (Krzyszton et al. 2022).

### Chromatin immunoprecipitation (ChIP)

Chromatin was isolated from dry, nondormant mature seeds and after 1, 3, and 5 days of secondary dormancy induction for WT and *brm-1/BRM-GFP* lines (Jarończyk et al. 2021). ChIP was performed as described previously (Kowalczyk 2017) with some modifications. The 60 mg of frozen seeds were ground to a powder using a pestle and mortar and suspended in 10 ml of MC buffer (0.1 m sucrose; 10 mm sodium phosphate pH 7; 50 mm NaCl). Then, 37% formaldehyde was added to the final concentration of 1% and samples were mixed softly on a rotating wheel for 10 min at 4 °C. After mixing, 625 *µ*l of 2 m glycine was added and samples were rotated for another 10 min. Then, the samples were filtered through a double layer of Miracloth Quick Filtration Material, and centrifuged for 10 min at 1,500 × *g* at 4 °C. After centrifugation, samples were resuspended in 5 ml of Honda buffer (0.44 m sucrose; 1.25% Ficoll; 2.5% Dextran T40; 20 mm HEPES-KOH pH 7.4; 0.5 m EDTA; 0.5% Triton X-100; 10 mm β-Mercaptoethanol and freshly added 0.0005 m PMSF and 1×Complete EDTA-free protease inhibitor) and spun for 10 min at 1,800 × *g* at 4 °C. The nuclear pellet was resuspended in 500 *µ*l ChIP Lysis/sonication buffer (50 mm HEPES-KOH pH 7.4; 150 mm NaCl; 1 mm EDTA; 1% Triton X-100; 0.8% SDS; 10 mm β-Mercaptoethanol and freshly added 0.0005 m PMSF and 1×Complete EDTA-free protease inhibitor) and sonicated twice 30s-ON/30s-OFF for 25 min using Bioruptor Sonication System (Diagenode). 1/10 of each sample was saved as input control and 20 *µ*l for sonication control. Sonication efficiency was verified by running decrosslinked samples on 1% agarose gel. GFP-Trap Agarose beads were prepared according to the manufacturer's protocol (Chromotek). The lysates of sonicated samples were added to equilibrated beads and placed on a rotating wheel in a cold room for 2 to 4 h. After incubation, beads were centrifuged for 2,500 × *g* for 5 min at 4 °C and washed twice for 5 min with 1 ml of low salt wash buffer (150 mm NaCl; 1% Triton X-100; 2 mm EDTA; 20 mm Tris pH 8.0; 0.1% SDS). Then, beads were washed for 5 min with 1 ml of high salt wash buffer (500 mm NaCl; 1% Triton X-100; 2 mm EDTA; 20 mm Tris pH 8.0; 0.1% SDS) and centrifuged for 2,500 × *g* for 5 min at 4 °C. Then, 500 *µ*l of phenol:chloroform:isoamyl alcohol mixture (25:24:1; pH 8.0) were added and the samples were shaken for 10 min at 22 °C. After centrifugation for 10 min at 14,000 × *g*, the upper aqueous layers were collected and 0.1 volume of 3 m sodium acetate (pH 5.2), 1 *µ*l of glycogen (Thermo Fisher Scientific), and 1 ml 96% ethanol were added. The mixed samples were held at −80 °C for >1 h and then centrifuged for 30 min at 14,000 × *g* at RT. The pellets were washed with cold 70% ethanol, air-dried, and suspended in water. For the quantification of DNA fragments, samples were tested by qPCR. The sequences of all primers are given in Supplementary Table S2 or published by Dolata et al. (2015).

### Formaldehyde-assisted isolation of regulatory DNA elements

The FAIRE method was performed according to the published protocol (Omidbakhshfard et al. 2014) with minor modifications. For nuclei isolation, 100 mg of the dry and secondary dormancy-induced seeds of Col-0 and *brm-3* were used. Chromatin was sheared by sonication and the sonication efficiency was checked by electrophoresis in agarose gel as previously described for the ChIP protocol. To separate NDR (nucleosome-depleted regions), the sheared chromatin DNA was extracted by the PCI (phenol:chloroform:isopropanol) method. The enrichment was assessed by qPCR using primers indicated in Supplementary Table S2. Calculations were performed using the ΔΔ*Ct* method with normalization of a crosslinked sample (FAIRE) to noncrosslinked sample (UNFAIRE) as described in Omidbakhshfard et al. (2014) and then to *PP2A*—AT1G13320 (Supplementary Table S2) as an internal control.

## Metabolome analysis

### Sample preparation

Mature postharvested seeds (50 mg) in biological triplicates were ground with metal beads for 2× 90 s on a Tissue Lyser (Qiagen) at 30 Hz in 1.5 ml of cold (−20 °C) methanol spiked with an internal standard of deuterium-labeled abscisic acid (^2^H_6_ ABA, 0.2 *µ*g ml^−1^). Samples were shaken for 10 min at RT, and centrifuged for 5 min at 13,000 rpm, RT. The supernatant was transferred to a glass vial and the extract was dried with a SpeedVac concentrator (Savant SPD121P, Thermo Fisher Scientific) at RT. The pellets were extracted twice with 1.5 ml methanol, shaken, centrifuged, and collected in the same glass vial to be evaporated. After this, dry samples were solubilized in 100 *µ*l of methanol.

### Nontargeted metabolites analysis

Extracts were analyzed by liquid chromatography coupled to high-resolution mass spectrometry (LC-HRMS) using an UltiMate 3000 UHPLC system (Thermo Fisher Scientific) coupled to the ImpactII (Bruker) high-resolution Quadrupole Time-of-Flight (QTOF) mass spectrometry according to Villette et al. (2018) and Graindorge et al. (2022). Chromatographic separation was performed on an Acquity UPLC HSS T3 C18 column (2.1 × 100 mm, 1.8 *µ*m, Waters) coupled to an Acquity UPLC HSS T3 C18 pre-column (2.1 × 5 mm, 1.8 *µ*m, Waters) using a gradient of solvents A (H_2_O, 0.1% formic acid) and B (methanol, 0.1% formic acid). Chromatography was carried out at 35 °C, at a flux of 0.3 ml min^−1^, starting with 5% B for 2 min, reaching 100% B at 10 min, holding 100% B for 3 min, and coming back to 5% B in 2 min (runtime 15 min). Samples were kept at 4 °C, 5 *μ*L were injected in a full loop mode with a washing step after sample injection with 150 *μ*l of wash solution (H_2_O/MeOH, 90/10, v/v). The spectrometer was equipped with an electrospray ionization (ESI) source and operated in positive and negative ion modes on a mass range from 20 to 1,000 Da with a spectra rate of 8 Hz in Auto MS/MS fragmentation mode. The end plate offset was set at 500 V, the capillary voltage set at 2.5 kV, the nebulizer at 29 psi, the dry gas at 8 l min^−1^, and the dry temperature of 200 °C. The transfer time was set at 20 to 70 *μ*s (positive mode) and 40.8 to 143 *μ*s (negative mode) and the MS/MS collision energy was at 80% to 120% with a timing of 50% to 50% for both parameters. The MS/MS cycle time was set to 3 s, the absolute threshold to 816 cts, and active exclusion was used with an exclusion threshold at 3 spectra, release after 1 min, and the precursor ion was reconsidered if the ratio current intensity/previous intensity was higher than 5. A calibration segment was included at the beginning of the runs allowing the injection of a calibration solution from 0.05 to 0.25 min. The calibration solution used was a fresh mix of 50 ml isopropanol/water (50/50, v/v), 500 *μ*l NaOH 1 m, 75 *μ*l acetic acid, and 25 *μ*l formic acid. The spectrometer was calibrated on the [M+H]^+^/[M−H]^−^ form of reference ions (57 masses from *m*/*z* 22.9892 to *m*/*z* 990.9196 in positive mode; 49 masses from *m*/*z* 44.9971 to *m*/*z* 996.8221 in negative mode) in high-precision calibration (HPC) mode with a standard deviation below 1 ppm before the injections for each polarity mode, and re-calibration of each raw data was performed after injection using the calibration segment. Molecular features were processed with MetaboScape version 4.0 (Bruker, Bremen, Germany). Molecular features were considered and grouped into buckets containing one or several adducts and isotopes from the detected ions with their retention time and MS/MS information when available. The parameters used for bucketing are a minimum intensity threshold of 10,000 (positive mode) or 1,000 (negative mode), a minimum peak length of 3 spectra, a signal-to-noise ratio (S/N) of 3, and a correlation coefficient threshold set at 0.8. The [M+H]^+^, [M+Na]^+^, [M+K]^+^, and [M+NH_4_]^+^ ions (positive mode); [M−H]^−^ and [M+Cl]^−^ ion (negative mode) were authorized as possible primary and seed ions. Replicate samples were grouped and only the features found in 80% of the samples of one group were extracted from the raw data. The obtained lists of features from positive and negative ion modes were merged. The parameters used for metabolite annotation were as follows. The maximum allowed variation on the mass (Δ*m*/*z*) was set to 3 ppm, and the maximum mSigma value (assessing the good fitting of isotopic patterns) was set to 30. The merged list of features was annotated using SmartFormula to generate a raw formula based on the exact mass of the primary ions and the isotopic pattern. Analyte lists were derived from KNApSAcK (http://www.knapsackfamily.com/KNApSAcK_Family/), PlantCyc (https://plantcyc.org/), FooDB (http://foodb.ca), LipidMaps (https://www.lipidmaps.org/), and SwissLipids (https://www.swisslipids.org/) to obtain a level 3 annotation according to Schymanski classification (tentative candidates based on exact mass and isotopic profile) (Schymanski et al. 2014). Spectral libraries (Bruker MetaboBASE Personal Library 3.0, MoNA_LCMSMS_spectra, MSDIAL_LipidBDs-VS34) were searched to obtain level 2 annotations (probable structure based on library spectrum match (MS2 data) according to Schymanski et al. (2014)). PubChem IDs, SMILES, and InChiKeys were obtained from PubChem (https://pubchem.ncbi.nlm.nih.gov/idexchange/idexchange.cgi) for chemical enrichment studies using ChemRICH tool (Barupal and Fiehn 2017).

### Sugar determination

Soluble sugar contents were assessed by HPLC (Dionex) according to Rosnoblet et al. (2007). Analysis was performed on 4 replicates of 15 mg of mature *Arabidopsis* seeds. In brief, seeds were ground in a mortar in the presence of 1 ml 80% methanol containing melizitose as the internal sugar standard. After heating at 76 °C for 15 min, the liquid was evaporated under vacuum. The residue was dissolved in 1 ml distilled water and centrifuged for 1 min at 13,000 × *g*. Sugars were analyzed by HPLC on a Carbopac PA-1 column (Dionex Corp.) (Rosnoblet et al. 2007).

### Targeted hormone analysis

Auxin, ABA, and GA were analyzed by ultra-high-performance liquid chromatography (UHPLC) on an UltiMate 3000 UHPLC system (Thermo Fisher Scientific)coupled to EvoQ Elite (Bruker) mass spectrometer equipped with an ESI source in MS/MS mode as described in Zumsteg et al. (2023).

### Statistical analyses

Statistical tests were done using a Student–Newman–Keuls test or a two-tailed *t*-test, implemented in Microsoft Office Excel.

### Accession numbers

Sequence data from this article can be found in the GenBank/EMBL data libraries under accession numbers listed in Supplementary Tables S1 and S2.

## Supplementary Material

kiae642_Supplementary_Data

## Data Availability

The 3′RNA-seq data generated for this study have been deposited at the Gene Expression Omnibus (GEO) under the accession code GSE251921.

## Supplementary data

The following materials are available in the online version of this article.

**Supplementary Figure S1.** Untargeted metabolite analysis of mature seeds of *brm3*, *dog1-4*, and *brm3 dog1-4*.

**Supplementary Figure S2.** Heatmap showing significant and differential transcript levels of genes of selected pathways.

**Supplementary Figure S3.** HPLC quantification of hormones in dry mature seeds of mutants compared to Col-0 WT.

**Supplementary Figure S4.** UHPLC quantification of total soluble sugars in dry mature seeds of mutants compared to Col-0 WT.

**Supplementary Figure S5.** Glutathione biosynthesis pathway regulation by BRM is *DOG1* gene-dependent.

**Supplementary Figure S5.** Seed physiology is affected in *brm3* mutants compared to the WT.

**Supplementary Figure S6.** Secondary seed dormancy phenotype of the selected mutants.

**Supplementary Figure S7.** Inactivation of SWP73A—BAS SWI/SNF specific subunit—results in stronger dormancy, higher *DOG1* expression, and decreased expression of *asDOG1*.

**Supplementary Figure S8.** ChIP-qPCR on BRM binding to *DOG1* locus.

**Supplementary Figure S10.** RT-qPCR expression analysis of the *DOG1* mRNA level.

**Supplementary Figure S11.** RT-qPCR analysis of selected ABA and GA marker genes during secondary dormancy induction.

**Supplementary Figure S12.** RT-qPCR analysis of selected ABA and GA marker genes in *dog1-4* mutant.

**Supplementary Figure S13.** RT-qPCR expression analysis of the native *DOG1* vs *LUC::DOG1* transgene with *dTATA* mutations in antisense region of independent transgenic lines.

**Supplementary Figure S14.** Primary seed dormancy phenotype of *3xbrd*, *brm-3*, *dog1-4*, and double *brm-3dog1-4* mutants in comparison to Col-0 WT seeds.

**Supplementary Figure S15.**  *DOG1* gene scheme and splicing analysis.

**Supplementary Table S1.** GO terms and ChemRICH metabolite analyses.

**Supplementary Table S2.** List of all primers used in this study.

## Dive Curated Terms

The following phenotypic, genotypic, and functional terms are of significance to the work described in this paper:

GSTU10 Gramene: AT1G74590
GSTU10 Araport: AT1G74590

MYB122 Gramene: AT1G74080
MYB122 Araport: AT1G74080

ABI3 Gramene: AT3G24650
ABI3 Araport: AT3G24650

AFP2 Gramene: AT1G13740
AFP2 Araport: AT1G13740

BRM Gramene: AT2G46020
BRM Araport: AT2G46020

MYB34 Gramene: AT5G60890
MYB34 Araport: AT5G60890

MYB51 Gramene: AT1G18570
MYB51 Araport: AT1G18570

RGL3 Gramene: AT5G17490
RGL3 Araport: AT5G17490

cyp707a1 Gramene: AT4G19230
cyp707a1 Araport: AT4G19230

ABI5 Gramene: AT2G36270
ABI5 Araport: AT2G36270

DOG1 Gramene: LOC_Os02g44250
DOG1 Araport: LOC_Os02g44250

GAI Gramene: AT1G14920
GAI Araport: AT1G14920

RGL1 Gramene: AT1G66350
RGL1 Araport: AT1G66350

RGL2 Gramene: AT3G03450
RGL2 Araport: AT3G03450

NCED4 Gramene: AT4G19170
NCED4 Araport: AT4G19170

cyp707a2 Gramene: Solyc08g005610
cyp707a2 Araport: Solyc08g005610

CYP79B3 Gramene: AT2G22330
CYP79B3 Araport: AT2G22330

ATP CHEBI: CHEBI:15422

EDTA Gramene: Ethylene Diamine Tetraacetic Acid
EDTA Araport: Ethylene Diamine Tetraacetic Acid
