# Supplementary material for: The BRAHMA-associated SWI/SNF chromatin remodeling complex controls Arabidopsis seed quality and physiology
Source: Plant Physiol. 2024 Dec 11;197(1):kiae642. doi: 10.1093/plphys/kiae642 (PMC11668257; doi:10.1093/plphys/kiae642)
Supplement: kiae642_Supplementary_Data [file kiae642_supplementary_data.zip › PP2024RA01076R1_Supplemental_Figures_1_15_UPDATED12.02.2024.pdf]

SUPPLEMENTARY FIGURES

The BRAHMA-associated SWI/SNF chromatin remodeling complex controls Arabidopsis seed quality and physiology

Wrona *et al.*, 2024

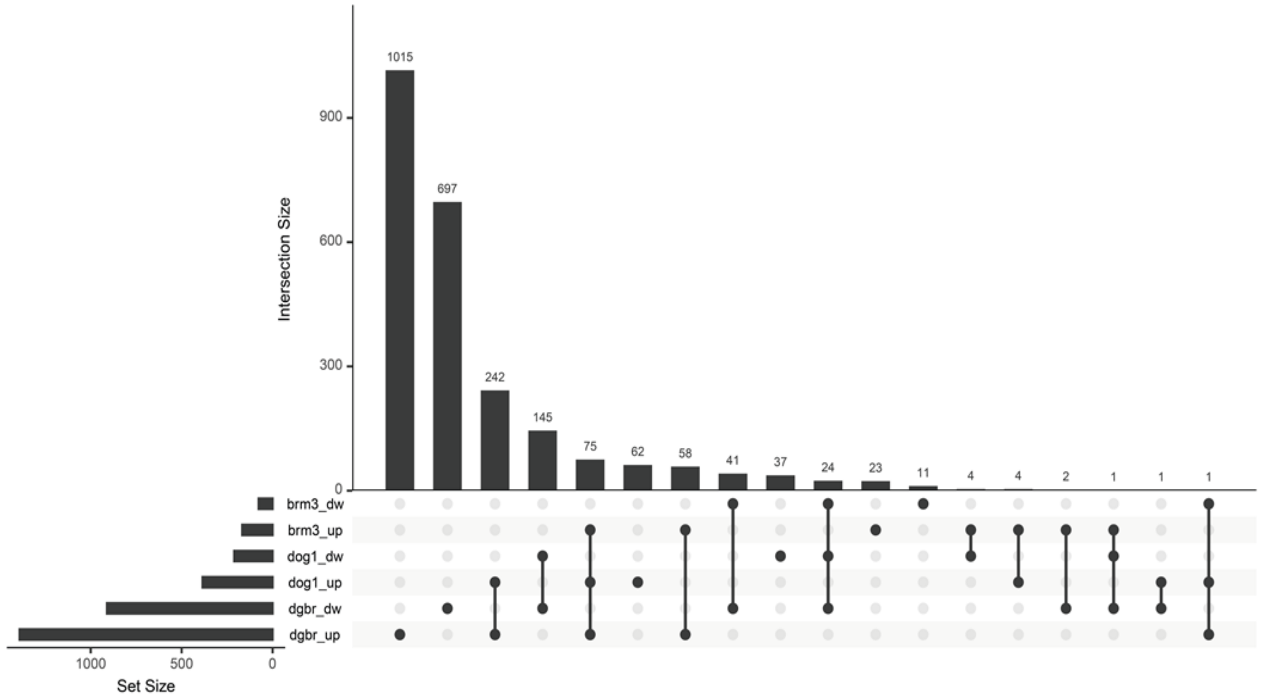

Supplementary Figure S1. Extended version of DEG analysis shown on figure 1B. Overlay between single *brm3*, *dog1-4* and double *brm3dog1-4* mutants of DOG1-dependent genes. the x-axis is Set. 3’RNA-seq, differential analysis was performed DESeq2 – genes with FDR < 0.05 and absolute fold change > 1 were considered as differentially expressed.

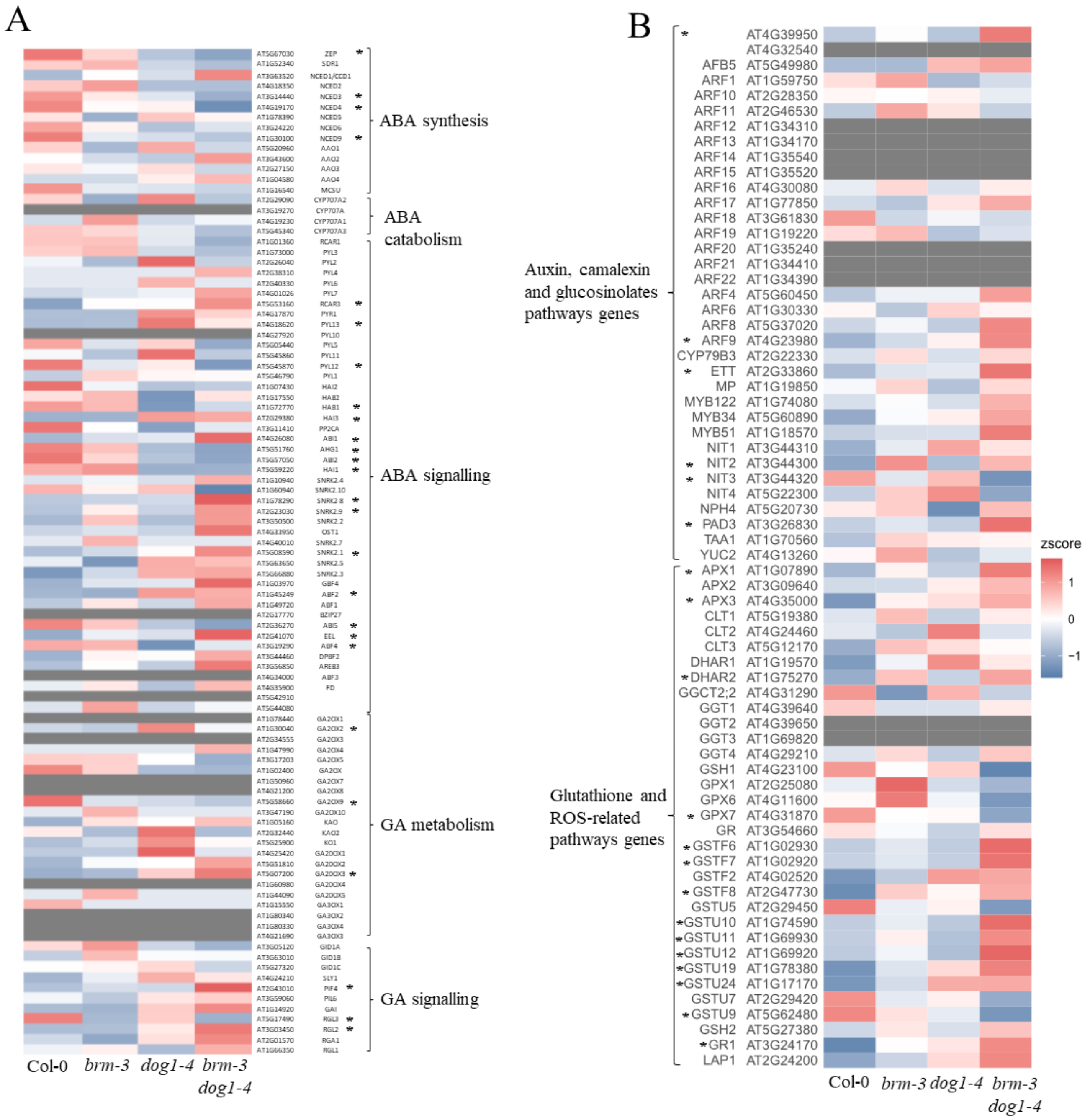

Supplementary Figure S2. Heatmap showing significant and differential transcript levels of genes of selected pathways.

(**A**) ABA and GA biosynthesis, catabolism, signaling and (**B**) auxin, camalexin, glutathione and glucosinolates pathway genes in dry seeds of Col-0 (WT), *brm-3*, *dog1-4* and *brm-3 dog1-4* mutant. Asterisk indicates a significantly different genes between *brm3*, *dog1-4* single, *brm-3 dog1-4* double mutants and *Col-0* (WT). 3'RNA-seq shown on panels A and B, differential analysis was performed using Seurat FindMarkers Wilcoxon Rank Sum test; adjusted P-value 0.05; DESeq2 – genes with FDR < 0.05 and absolute fold change > 1 were considered as differentially expressed.

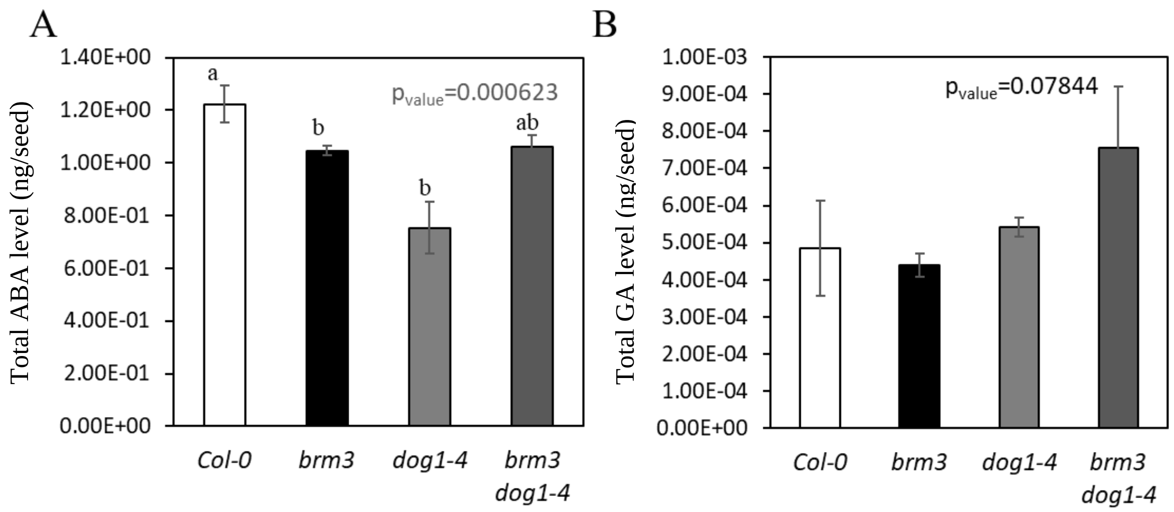

Supplementary Figure S3. HPLC quantification of hormones in dry mature seeds of mutants compared to Col-0 WT. (**A**) Total ABA and (**B**) GA content. Data are the mean (±SE) of seven to eight replicates of 50mg of dry mature seeds. When significantly different (ANOVA, $P<0.05$), values were ranked into groups as indicated by the respective letter using a Student-Newman-Keuls test.

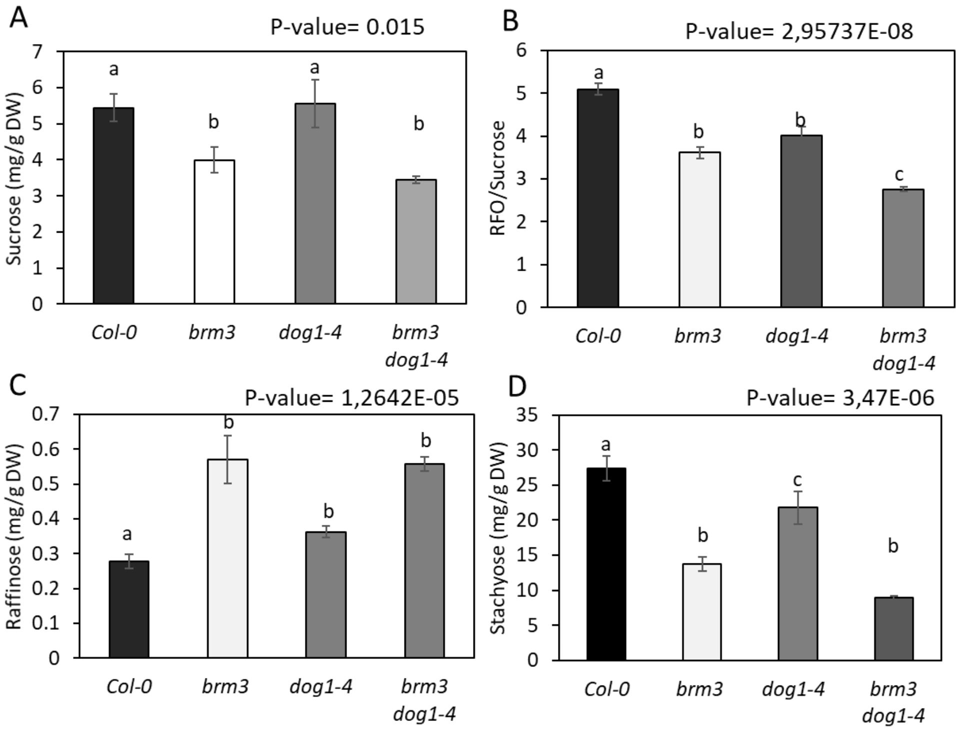

Supplementary Figure S4. UHPLC quantification of total soluble sugars in dry mature seeds of mutants compared to Col-0 WT. (**A**), sucrose; (**B**), RFO/sucrose; (**C**) Raffinose; (**D**) Stachyose. Data are the mean (±SE) of four replicates of 10mg of dry mature seeds. When significantly different (ANOVA, p<0.05), values were ranked into groups as indicated by the respective letter using a Student-Newman-Keuls test: letters indicate homogenous groups (P<0.05).

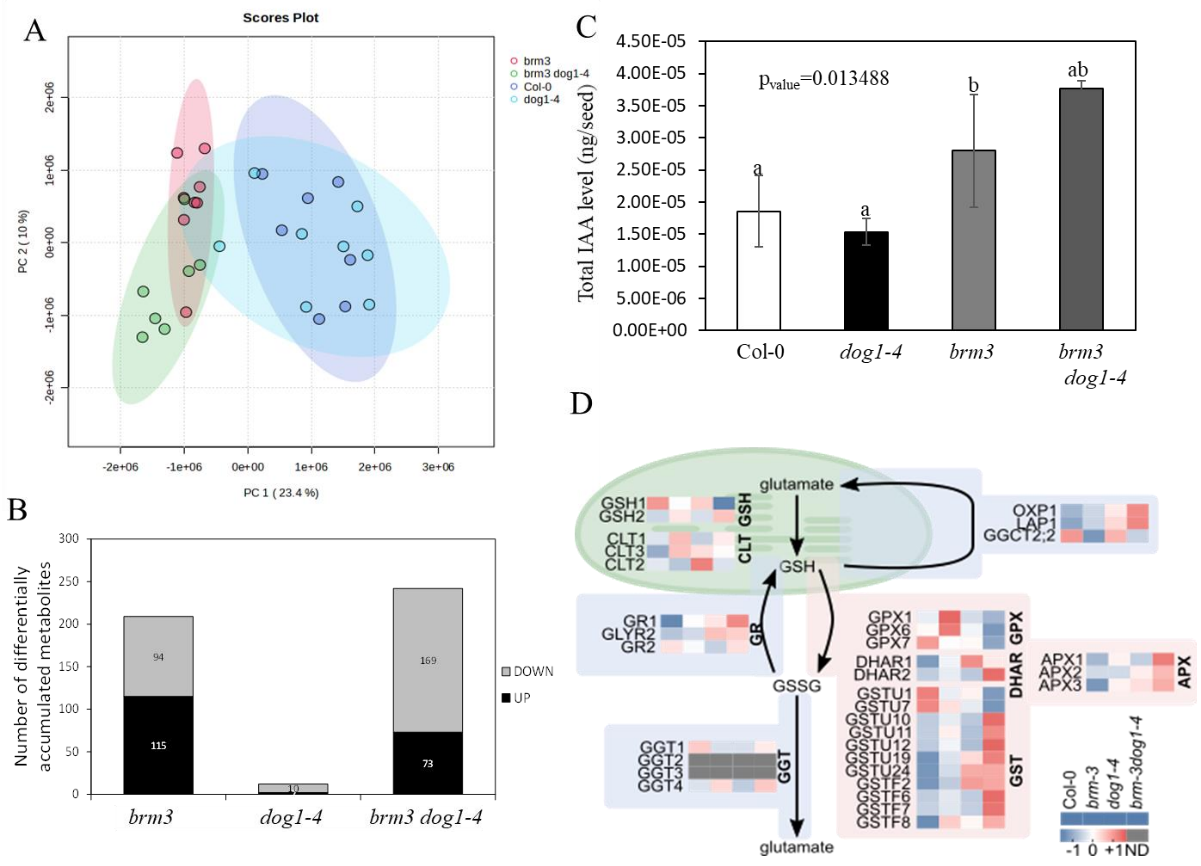

Supplementary Figure S5. Untargeted metabolite analysis, auxin content and glutathione pathway genes of mature seeds of the *brm3*, *dog1-4* and double *brm3dog1-4* mutants. (**A**) PCA analysis. Data represent seven to eight replicates of 50mg of dry mature seeds. (**B**) Number of metabolites changed in single and double mutants compared to wild type seeds, n=5; (**C**) HPLC quantification of total auxin (IAA) level in *brm3*, *dog1-4* and double *brm3dog1-4* mutants. Data are the mean (±SE) of seven replicates of 50mg of dry mature seeds. When significantly different (ANOVA, P<0.05), values were ranked into groups as indicated by the respective letter using a Student-Newman-Keuls test. (**D**) Glutathione biosynthesis pathway regulation by BRM is *DOG1* gene-dependent. Heatmaps show relative expression in Col-0, *brm-3*, *dog1-4*, *brm-3dog1-4* based on RNAseq of selected genes related to the glutathione metabolism and functions.

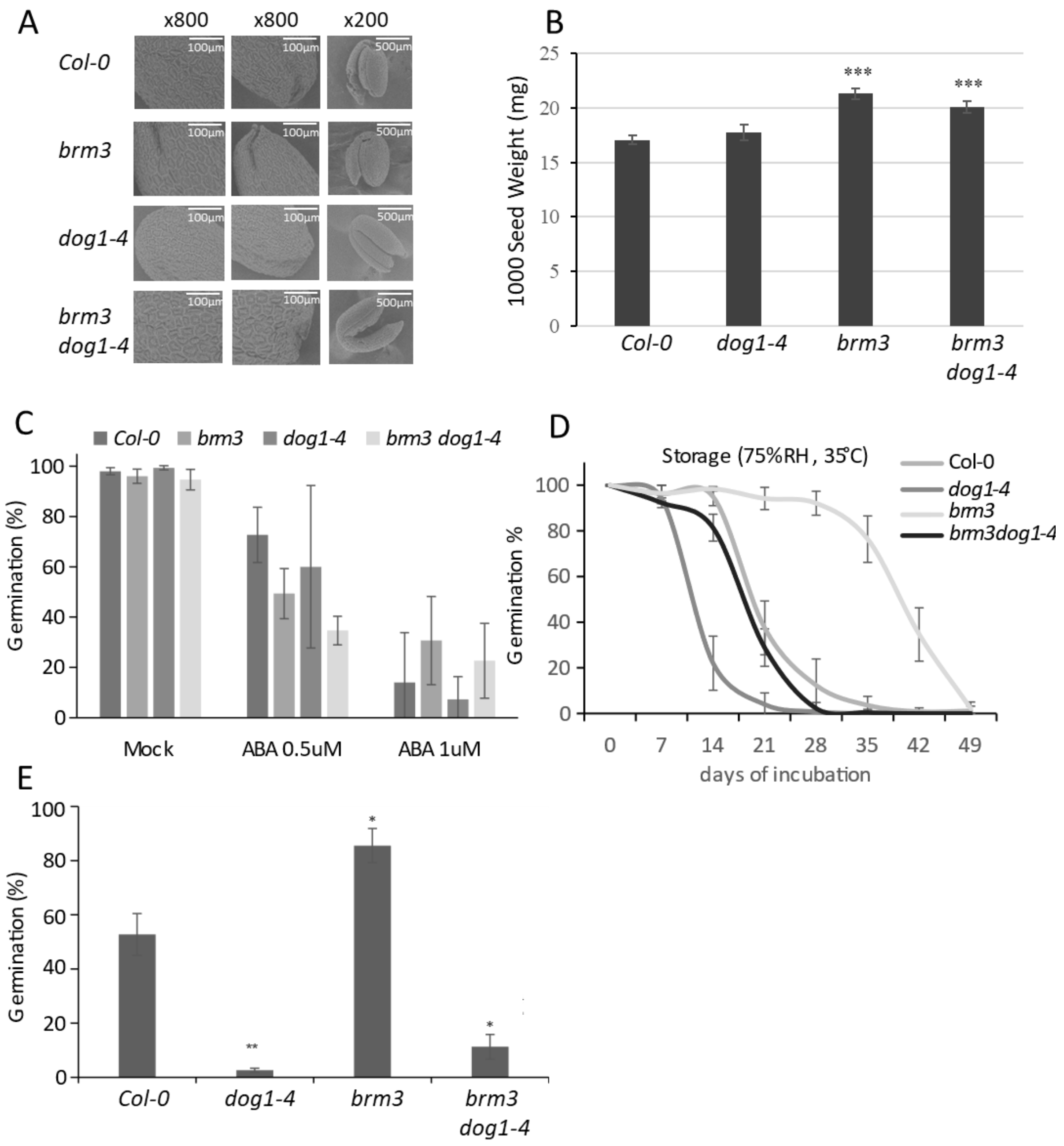

Supplementary Figure S6. Seed physiology is affected in *brm3* mutants compared to Col-0 wild type. (**A**) Seed size pictures. Note that several pictures are of the same seed as shown on Fig. 2A. (**B**) Seed weight of Col-0, *dog1-4*, *brm3*, *brm3 dog1-4* mutants. Data are the average (±SE) of four replicates of 1000 seeds. (**C**) ABA-induced 10 days germination analyses of *brm3*, *dog1-4* and double *brm3 dog1-4* mutant seeds compared to mock solution. Data are the average (±SE) of three replicates of 100 seeds. The x-axis shows mock, 0,5µM and 1 µM ABA concentrations. (**D**) Loss of viability during storage of indicated phenotype. Data are collected from one culture and are the average (±SE) of 3 replicates of 100 seeds. (**E**) Germination analysis of naturally aged Col-0, *dog1-4*, *brm3* and double *brm3 x dog1-4* mutant seeds stored for 4 years at normal conditions, n=4. Asterisks indicate significant differences compared to Col-0 wild type seeds for panels B and E (t-test, *, $p<0.05$; **, $p<0.01$ ***, $p<0.001$), error bars represent standard deviation (±SD).

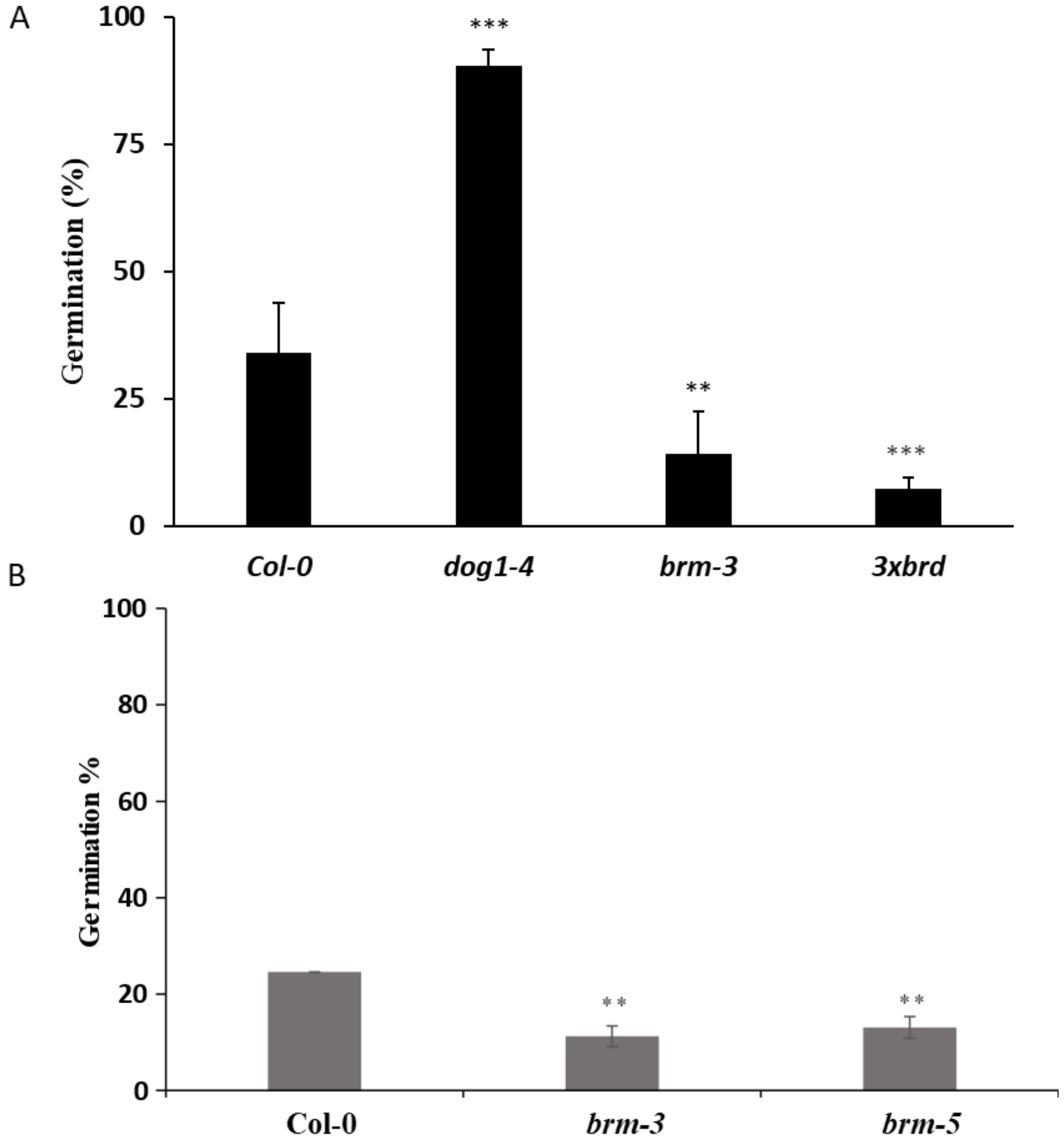

Supplementary Figure S7. Secondary seed dormancy phenotype of the selected mutants (A) *dog1-4, brm-3, 3xbrd and* (**B**) *brm-3* and *brm-5* in comparison to *Col-0* WT seeds from independent seeds harvests. Germination was scored after 7 days of SD induction and 3 days of normal growth. Asterisks indicate significant differences compared to *Col-0* wild type (t-test, **, $p<0.01$ and ***, $p<0.0001$, n=5, error bars represent standard deviation (±SD).

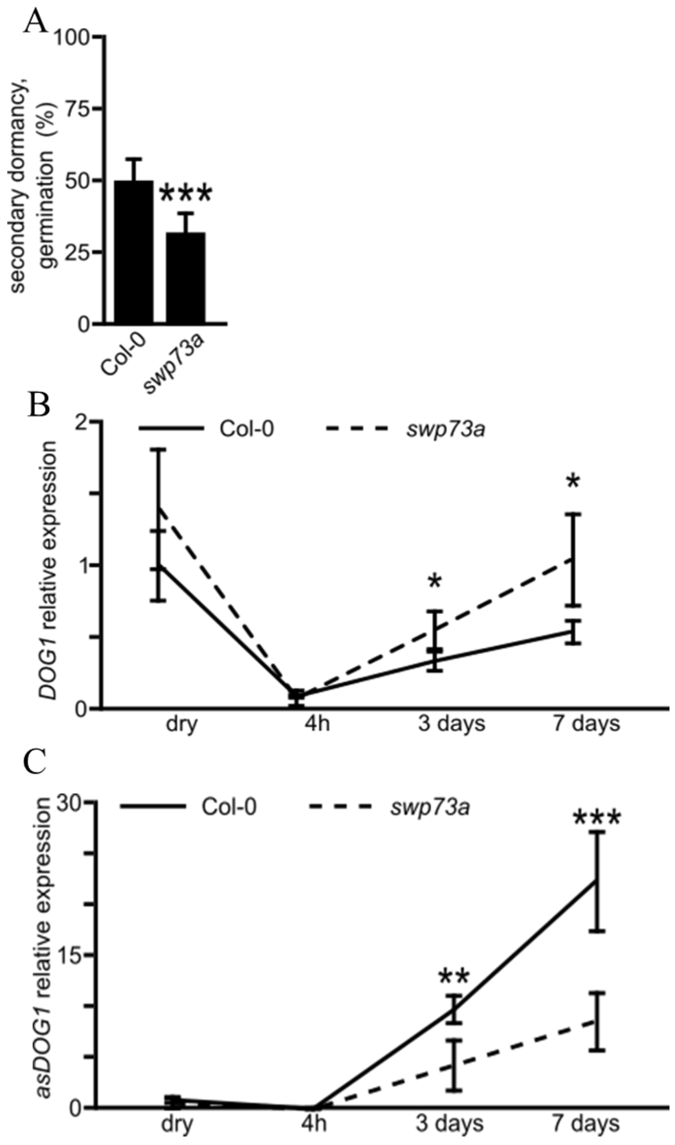

Supplementary Figure S8. Inactivation of SWP73A - BAS SWI/SNF specific subunit - results in stronger dormancy, higher *DOG1* expression and decreased expression of *asDOG1*. (**A**) Seed germination phenotypes of Col-0 WT and *swp73a* mutant after 7 days of secondary dormancy induction and 4 days at normal growth conditions; mRNA level of the (**B**) *DOG1* sense and (**C**) antisense DOG1 transcripts in the *swp73a* mutant vs Col-0 WT seeds under secondary dormancy inducing conditions (statistics apply to all panels, t-test, *, $P<0.05$, **, $P<0.01$ and ***, $P<0.0001$, n=4, error bars represent standard deviation (±SD).

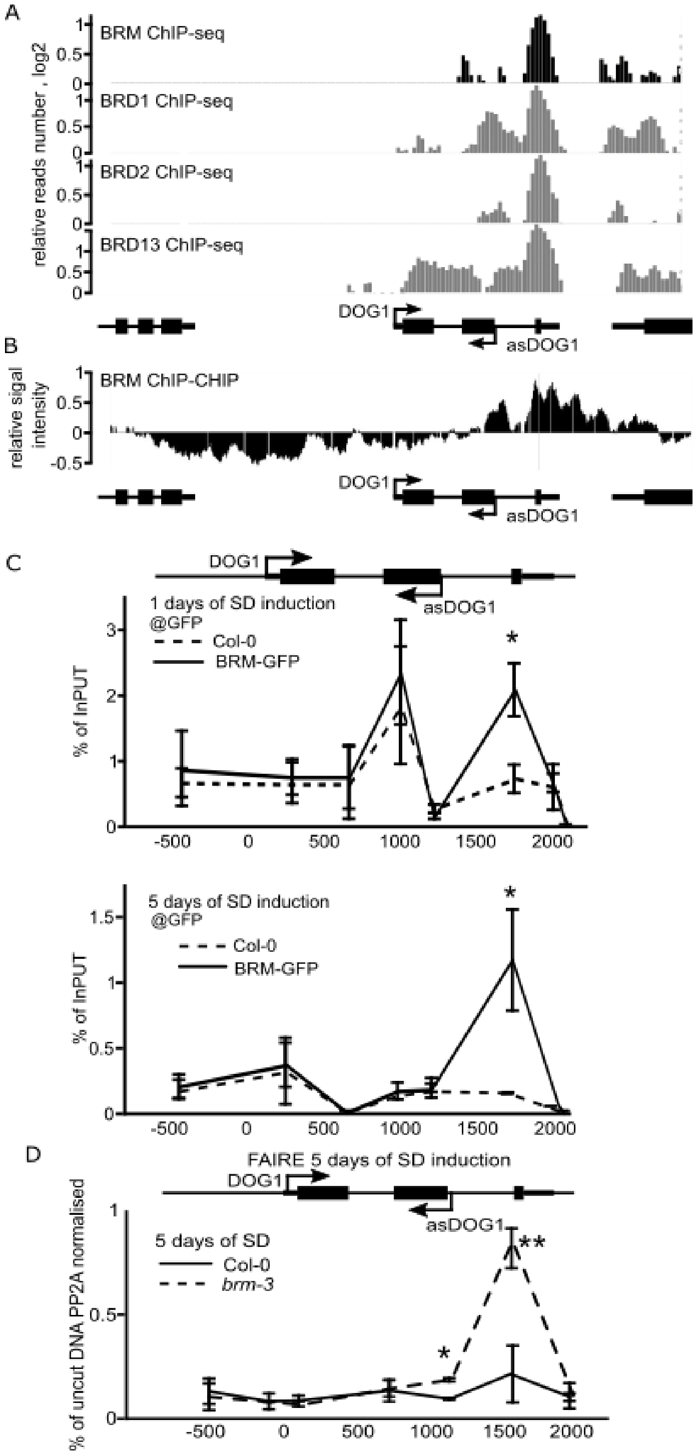

Supplementary Figure S9. ChIP-qPCR on BRM binding to *DOG1* locus. Genome wide ChIP seq data analysis on different sets of **(A)** BRM and BRD (Yu et al., 2020, 2021) and ChIP-ChIP data for (**B**) BRM (Archacki et al., 2016) binding within selected genomic locus including *DOG1* gene in *Arabidopsis* seedlings; (**C**) ChIP-qPCR BRM binding to *DOG1* experimentally

tested at day 1 and 5 in secondary dormancy induced seeds of Col-0 and BRM-GFP *brm-1* seeds using GFP antibodies. Percent of input normalized to PP2A gene region. (**D**) FAIRE-qPCR analysis of the Col-0 and *brm3* mutant seeds after 5 days of secondary dormancy. Percent of input normalized to UNFAIRE and PP2A gene region. Asterisk indicate significant differences compared to *Col-0* sample for panels C and D (t-test, *, P<0.05; **, P<0.01, n=4, error bars represent standard deviation (±SD)).

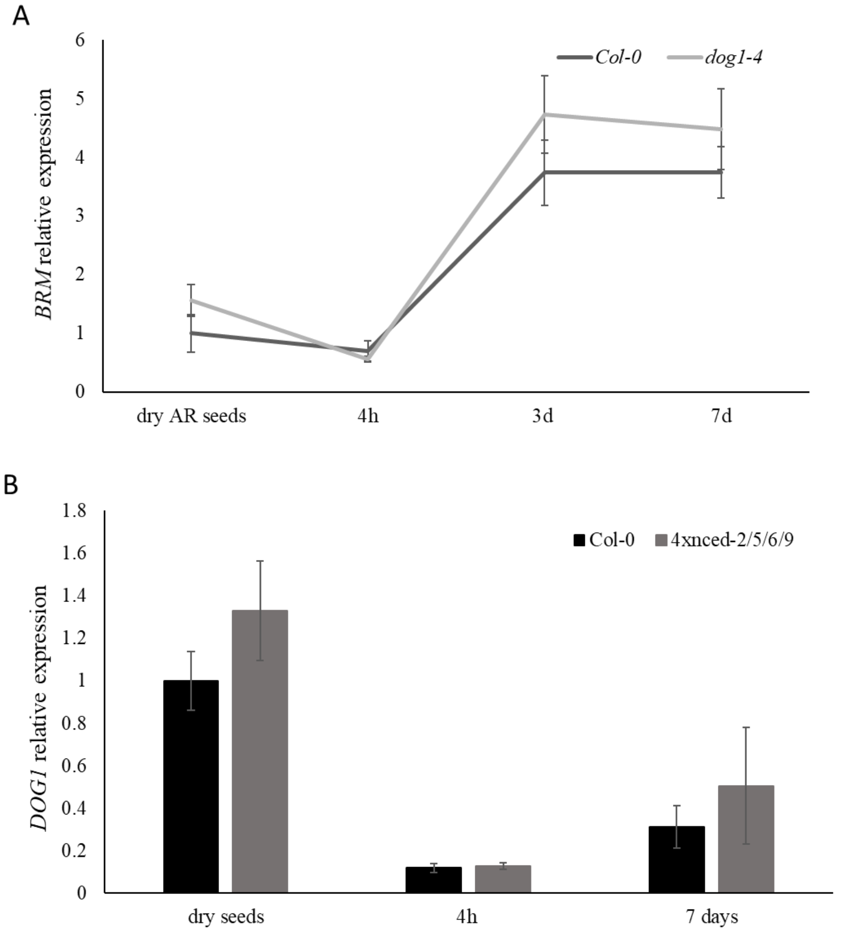

Supplementary Figure S10. RT-qPCR expression analysis of the *DOG1* mRNA level. (**A**) BRM mRNA level in Col-0 and *dog1-4* mutant under secondary dormancy inducing conditions, and (**B**) total *DOG1* mRNA level in Col-0 and quadruple *nced-2/5/6/9* mutant. The x-axis shows time/days of secondary dormancy induction (h-hours; d-days), n=4, error bars represent standard deviation (±SD) in all panels.

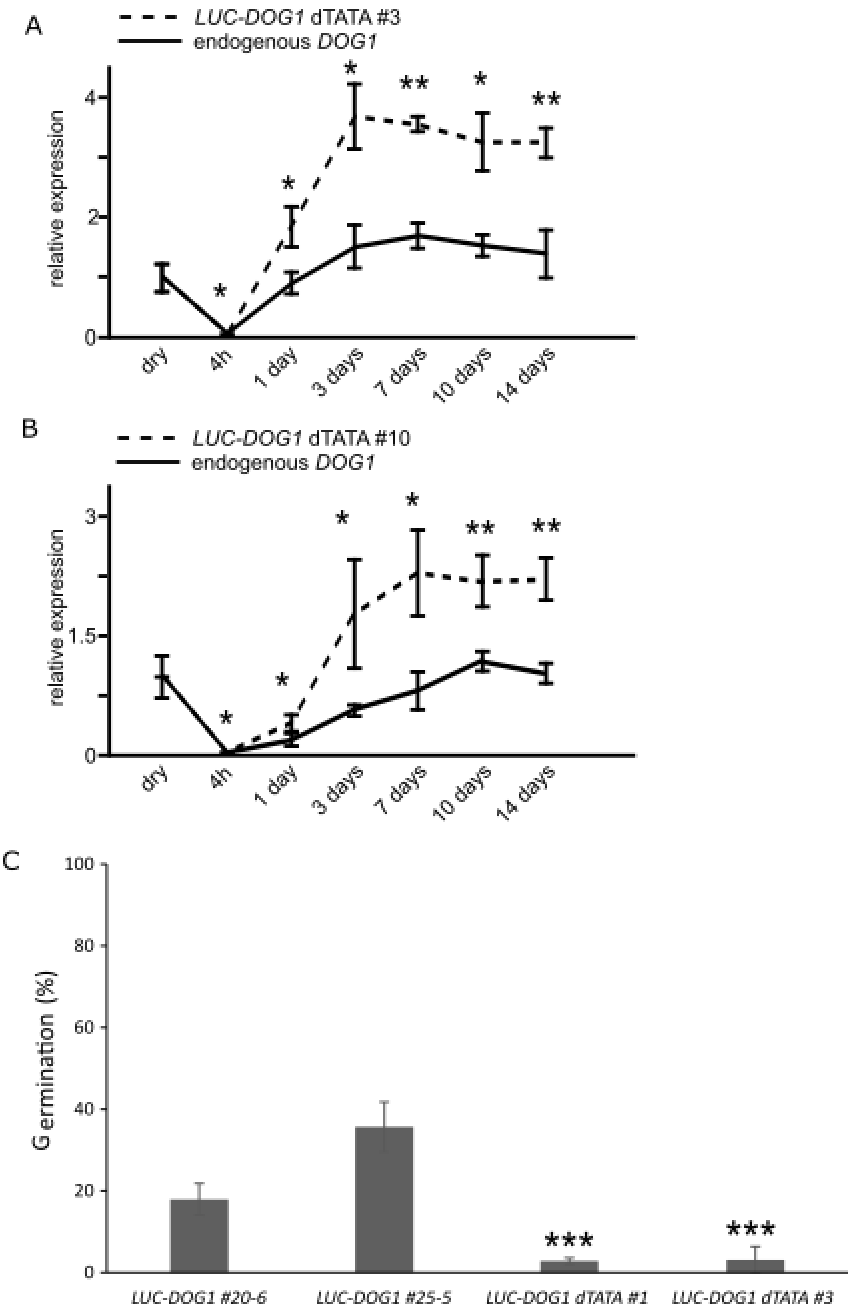

Supplementary Figure S11. RT-qPCR expression analysis of the native *DOG1* vs *LUC::DOG1* transgene with *dTATA* mutations in antisense region of independent transgenic lines #3 **(A)** and #10 **(B)** in secondary dormancy induced seeds; The x-axis on panels A and B shows time/days of secondary dormancy induction (h-hours; d-days); (**C**) Secondary seed dormancy phenotype of transgenic lines *LUC-DOG1* and *LUC-DOG1 dTATA.* The x-axis on panel C shows line numbers used in this study. Germination was scored after 7 days of SD induction and 3 days of normal growth. Asterisks indicate significant differences compared to Col-0 dry seeds (statistics apply to all panels, t-test, *, $P<0.05$, **, $P<0.01$ and ***, $P<0.0001$, $n=4$, error bars represent standard deviation (±SD).

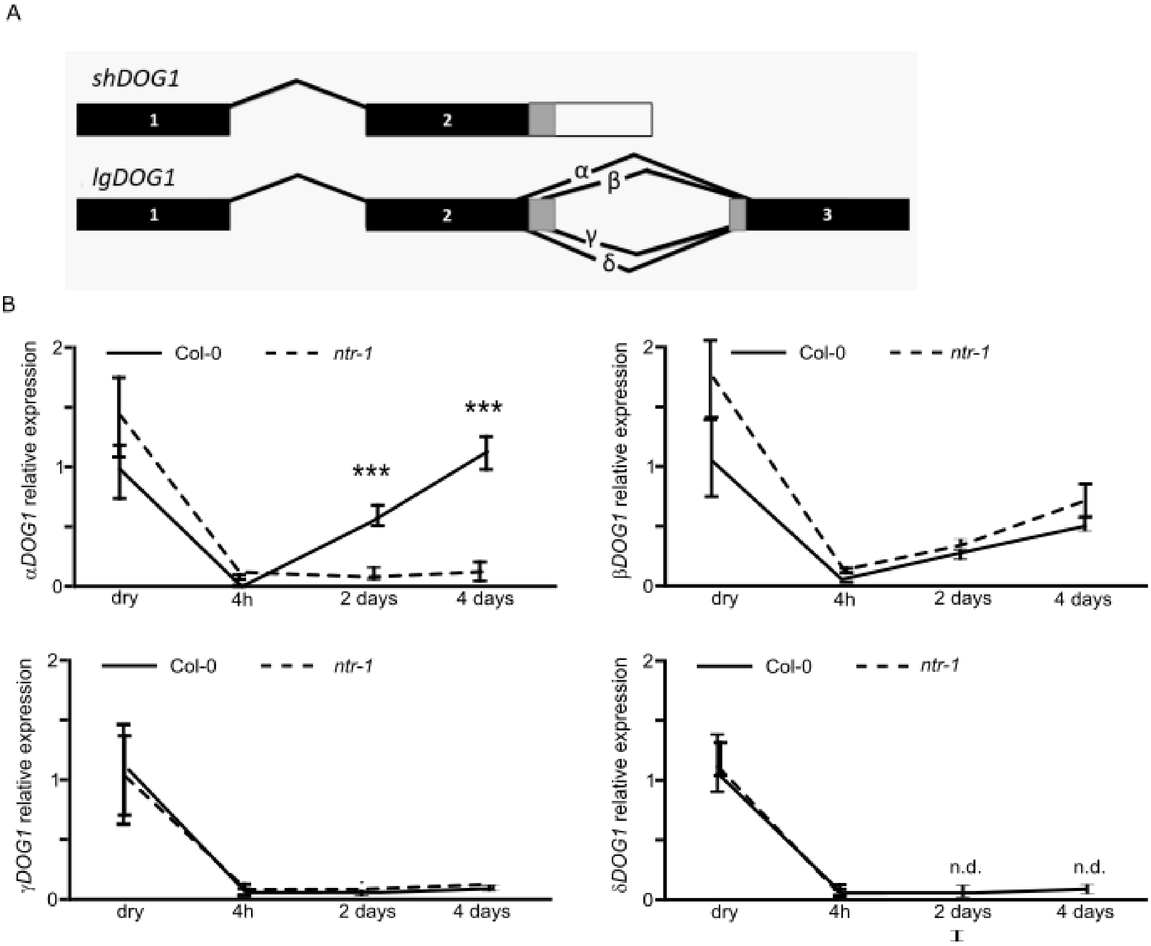

Supplementary Figure S12. *DOG1* gene scheme and splicing analysis; (A) *DOG1* gene scheme of different polyadenylation and splicing mRNA forms according to Cyrek et al., 2016, and **(B)** RT-qPCR expression analysis of *DOG1 α, β, γ, δ* - splicing mRNA forms between Col-0 WT and *ntr1* mutant seeds during secondary dormancy induction. The x-axis on panel B shows time/days of secondary dormancy induction (h-hours; d-days). Asterisks indicate significant differences compared to *Col-0* dry seeds (t-test, ***, P<0.0001, n=4, error bars represent standard deviation (±SD); n.d. – not detected *δDOG1* splicing mRNA form in the *ntr1* mutant at 2nd and 4th day of secondary dormancy induction.

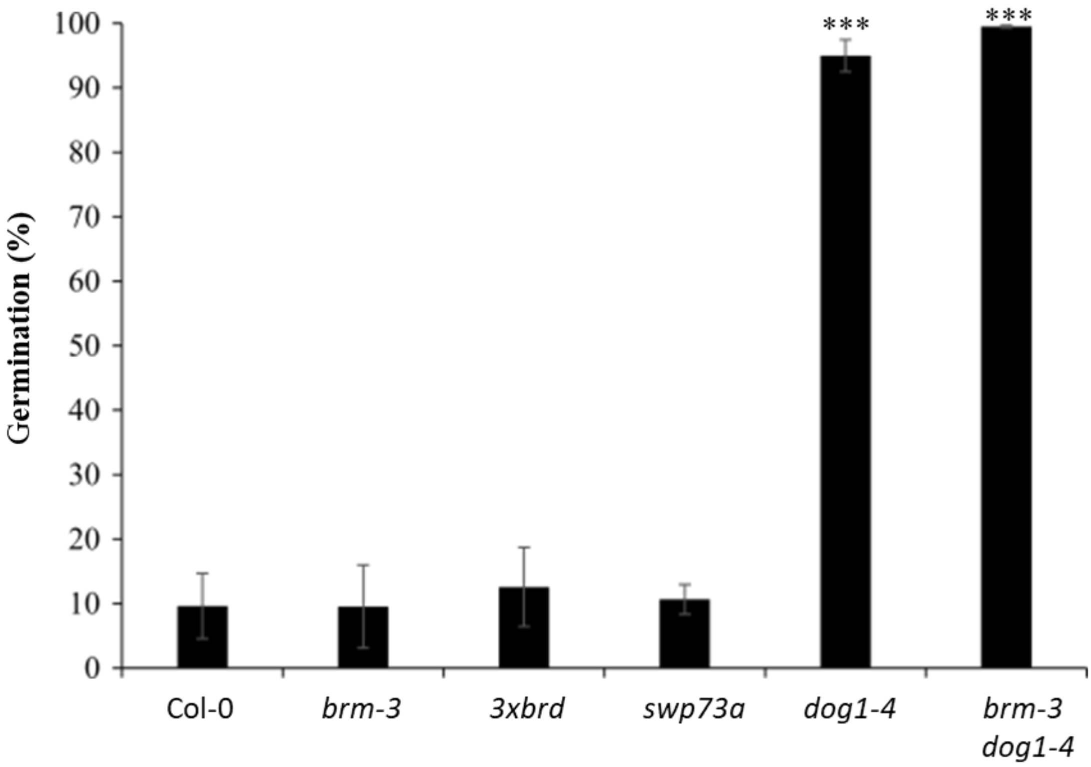

Supplementary Figure S13. Primary seed dormancy phenotype of *3xbrd, brm-3, dog1-4 and double brm-3dog1-4* mutants in comparison to *Col-0* WT seeds. Germination was scored after 7 days of normal growth. Asterisks indicate significant differences compared to Col-0 wild type (t-test, ***, P<0.0001, n=20, error bars represent standard deviation (±SD). Data represented here are summary of two independent experiments.

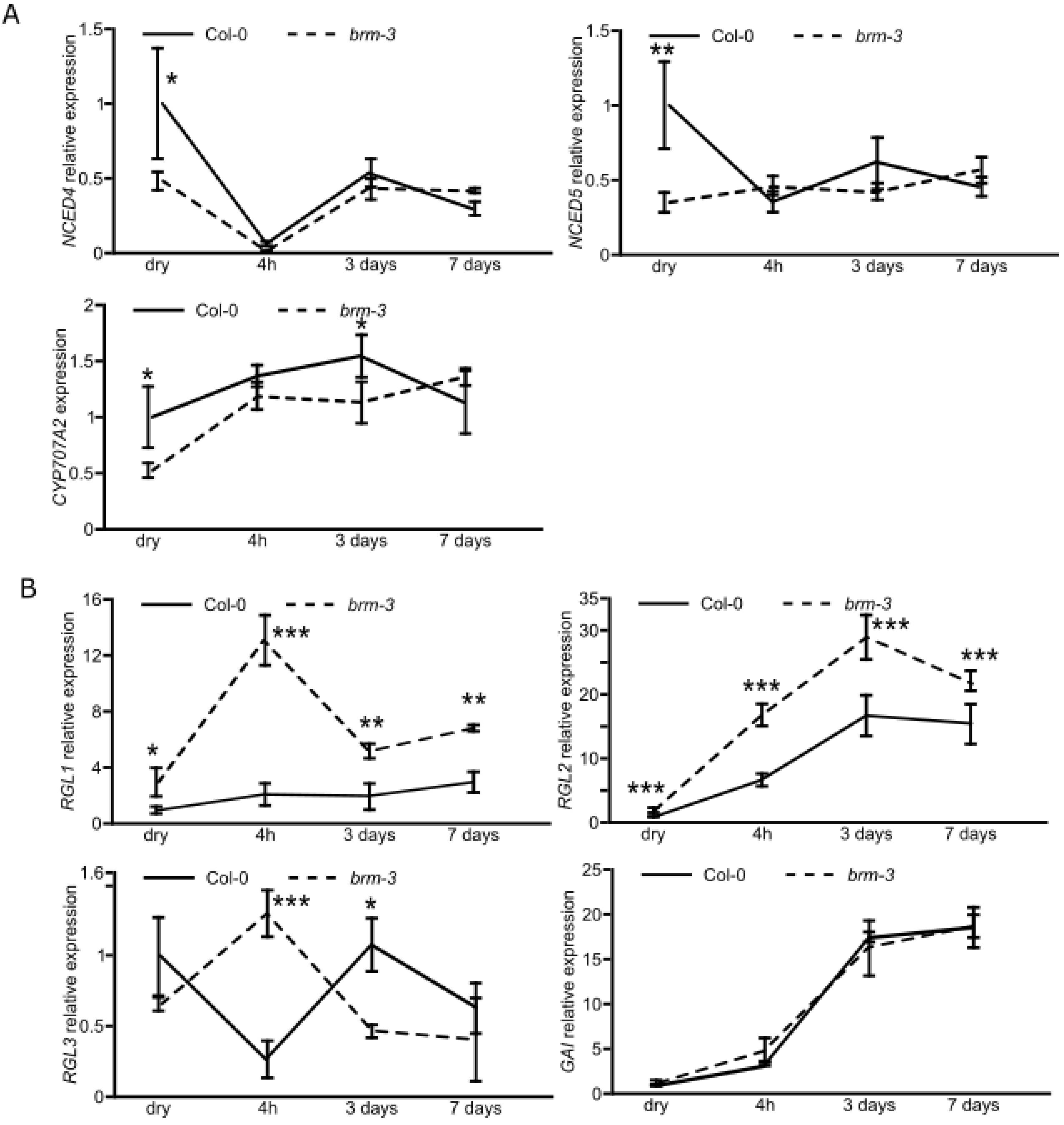

Supplementary Figure S14. RT-qPCR analysis of selected ABA and GA marker genes during secondary dormancy induction in *brm3* mutant. (A) ABA *(NCED4, NCED5, CYP707A2)* and **(B)** GA (*RGL1, RLG2, RGL3, GAI)* hormonal pathways marker genes in dry and secondary dormancy induced Arabidopsis Col-0 and *brm3* seeds. The x-axis on all panels shows time/days of secondary dormancy induction (h-hours; d-days). Asterisk indicate significant differences compared to *Col-0* (WT) seeds (t-test, *, P<0.05, **, P<0.01 and ***, P<0.0001, n=4, error bars represent standard deviation (±SD).

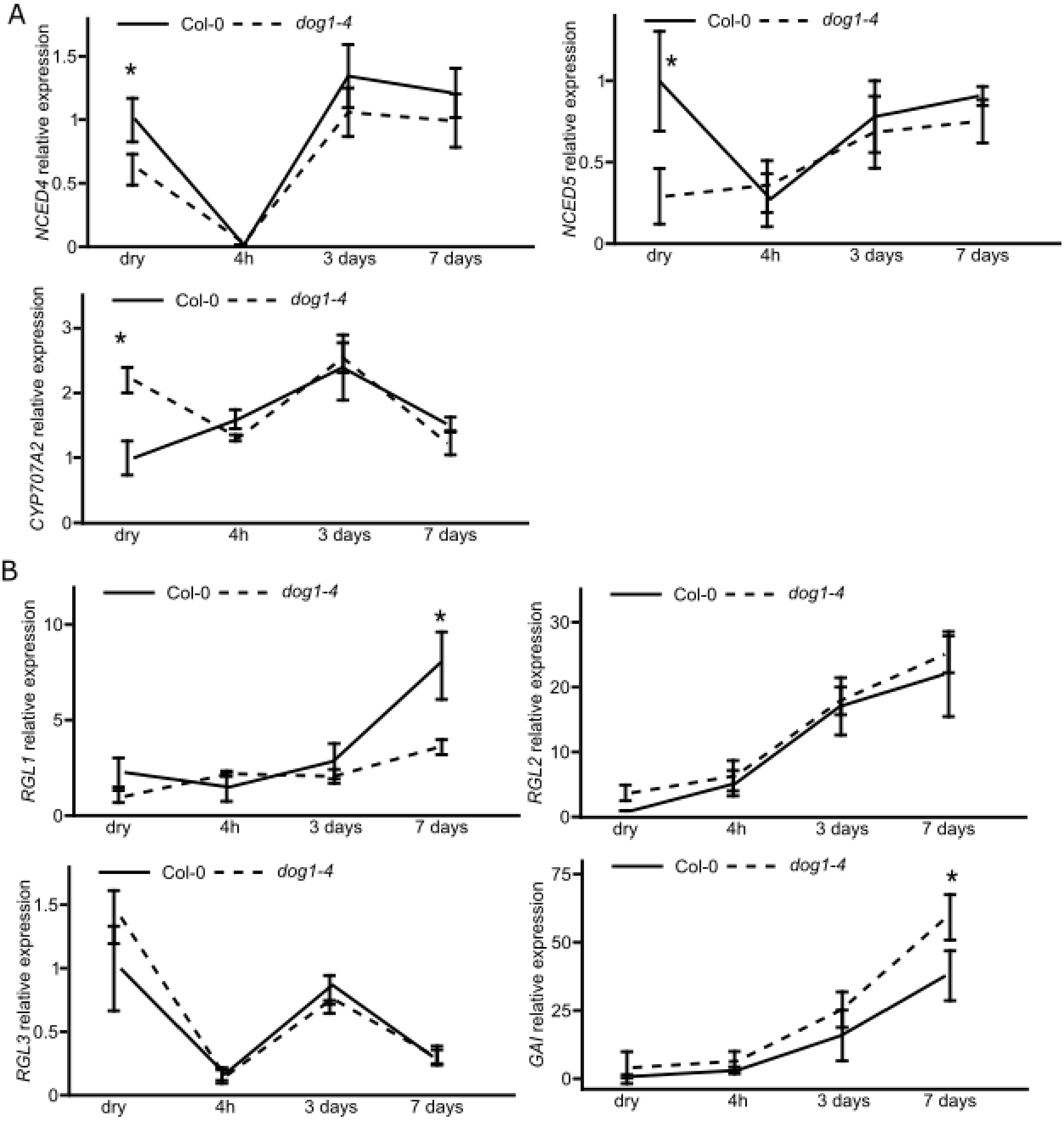

Supplementary Figure S15. RT-qPCR analysis of selected ABA and GA marker genes during secondary dormancy induction in *dog1-4* mutant. **(A)** ABA *(NCED4, NCED5, CYP707A2)* and **(B)** GA (*RGL1, RLG2, RGL3, GAI)* hormonal pathways marker genes in dry and secondary dormancy induced Arabidopsis Col-0 and *dog1-4* seeds. The x-axis on all panels shows time/days of secondary dormancy induction (h-hours; d-days). Asterisk indicate significant differences compared to *Col-0* (WT) seeds (t-test, *, $P<0.05$, $n=4$, error bars represent standard deviation (±SD).
