# Supplementary material for: The BRAHMA-associated SWI/SNF chromatin remodeling complex controls Arabidopsis seed quality and physiology
Source: Plant Physiol. 2024 Dec 11;197(1):kiae642. doi: 10.1093/plphys/kiae642 (PMC11668257; doi:10.1093/plphys/kiae642)
Supplement: kiae642_Supplementary_Data [file pp.01076.2024-s08.pdf › pp.01076.2024-s08.pdf]

Plant Physiology®

BRM-associated SWI/SNF complex controls Arabidopsis seeds quality and physiology

Magdalena Wrona, Julia Zinsmeister, Michal Krzyszton, Claire Villette, Julie Zumsteg, Pierre Mercier, Martine Neveu, Sebastian Sacharowski, Rafal Archacki, Boris Collet, Julia Buitink, Hubert Schaller, Szymon Swiezewski, and Ruslan Yatusevich

Review Timeline:

Submission Date:	15-Jul-2024
Editorial Decision:	13-Aug-2024
Revision Received:	17-Oct-2024
Accepted:	08-Nov-2024

13-Aug-2024

Dr. Szymon Swiezewski
Instytut Biochemii i Biofizyki Polskiej Akademii Nauk
Warsaw 02-106
Poland

RE: BRM-associated SWI/SNF complex controls Arabidopsis seeds quality and physiology

Dear Dr. Swiezewski:

Your manuscript has been reviewed by two experts in the field. The reviewers found your work intriguing, particularly noting the novelty of the proposed mechanism by which BRM controls DOG1 expression. However, both reviewers have highlighted several areas that require your attention for further improvement. Specifically, since the regulation through lncRNA asDOG1 transcripts is one of the major conclusions of this study. We encourage you to prioritize addressing comments related to the involvement of asDOG1 and to revise the manuscript to further substantiate this conclusion.

When you are ready to submit your revision, click the below link and find the red arrow under your Author Tasks heading for this revision submission.

If you want to visit this version of the manuscript, you may click the link below and then click on the folder that reads "Post Decision Manuscripts".

Link Not Available

When uploading the revised version of this article, please be sure to include as complete an answer as possible to the referees' criticisms during the first round of reviews in the "response to reviewers" section.

Plant Physiology considers a revision for up to 60 days after a manuscript has been provisionally accepted; limited extension of this timeframe is at the discretion of the Monitoring Editor. A reminder notice will be sent to you 2 weeks before the deadline, at which time you may choose to contact the editorial office to request an extension; if so, please provide a justification for your request. After the expiration of this deadline, the article would need to be submitted as a resubmission and would be handled as such.

Thank you very much for giving us an opportunity to review this work. I look forward to receiving the next version.

Sincerely,

Editor, Plant Physiology

Want to add this revision deadline to your calendar? Click below!

--- Reviewer comments:
Reviewer #1 (Comments for the Author):

This study revealed the involvement of BRM-associated SWI/SNF complex in the regulation of seed development and dormancy, and indicated that BRM regulates the process by cooperating with DOG1, a critical regulator of seed dormancy. The conclusion was supported by multiple methods, including genetic analysis, gene expression analysis, ChIP-PCR, and FAIRE. The study suggests a previously uncharacterized role in Arabidopsis, and demonstrates the underlying molecular mechanisms. However, I find that some experiments and analyses in the study need to be improved to confirm the conclusion.

Major points:
1. The results shown in Figure 1B were not correctly described in the text (line 316-318). The results did compared the

differentially expressed genes (DEGs) between brm-3 single mutant or dog1-4 single mutant and brm-3 dog1-4 double mutant but did not show a comparison of differentially expressed genes between the brm-3 and dog1-4 single mutants. Without the comparison of DEGs between brm-3 and dog1-4 single mutants, the relationship between the effects of brm-3 and dog1-4 on gene expression cannot be accurately evaluated.

2. The clustering of gene expression profiles are shown in Figure 1C. The information of the number of genes is important for the analysis. Without this information, it is difficult to determine to what extent the opposite effects of brm-3 and dog1-4 occur at the whole-genome level.

3. In line 343-346, related data described in the text should be indicated in the manuscript. Especially, the text indicated that 50 out of 244 genes are regulated by BRM in DOG1 dependent manner. This analysis was not shown.

4. The association of BRM with 3' end of DOG1 gene was indicated by ChIP-PCR. Because BRM ChIP-seq data have been shown in several previous study, whether the enrichment of BRM at the DOG1 locus is supported by these ChIP-seq data should be analyzed.

5. In Figure 3A and 3B, the direction of DOG1 gene and the position and direction of lncRNA within the DOG1 should be clearly marked. Otherwise, it is difficult to understand the results.

6. By comparing Figure 3A and 3B, the authors suggest that the BRM binding position shifts from exon 2 and exon 3 regions to exon 3. Although the enrichment of BRM at the exon 3 is significant, I feel that the enrichment change at the exon 2 between the two conditions is not convincing. The error bars of the ChIP-PCR values at the exon1 and exon 2 regions as shown in Figure 3B are too high to differentiate the change between the two conditions.

7. In line 535, "BRM supresses pAsDOG1 activity". Within this this statement "BRM" should be corrected to "brm", because BRM can actually activate the expression of pAsDOG1.

8. While the increase of DNA accessibility was enhanced under 5 days of SD compared to 3 days of SD, it is unclear whether the increase is caused by the change of BRM enrichment between the two conditions. The experiment will further contribute to understanding of the underlying molecular mechanisms.

9. Six positions were detected in FAIRE (Fig 4A, 4B), while seven positions were detected in ChIP-PCR (Fig 3A, 3B). Are the six positions used in FAIRE included in the seven positions used in ChIP-PCR? Because the FAIRE and ChIP-PCR data were compared in the text, this information should be clearly indicated.

10. The different splicing forms of DOG1 mRNAs were detected in the study. The different forms need to be indicated by schemes in Figures. Moreover, the full names of shDOG1 and lgDOG1 need to be shown.

Minor points:

1. In line 417, "that" should be corrected to "than".

2. According to the text, the order of Figure 3E and 3F should be converted.

Reviewer #2 (Comments for the Author):

BRM is putative component of SWI/SNF chromatin remodeling complex for gene epigenetic modification, and DOG1 is the main locus to determine primary seed dormancy, but their interaction was not full investigated before. In this manuscript, Wrona et al presented a series of subtle experiment to describe the novel function of BRM in control seeds quality and physiology, mainly in seed dormancy. Overall, this study is very interesting and expand our understanding on seed germination and dormancy process. Several concerns should be paid attention as below:

Main:
1) the mechanism of DOG1 in control secondary seed dormancy: it is known that DOG1 determine the primary seed dormancy, but the possible mechanism of DOG1 in control secondary seed dormancy is unknow, the author should provide more evidence and experiment to explain how DOG1 affect the seed dormancy, whether or how to affect ABA/GA level, starch metabolism or other possible pathway?

2) the seed longevity and secondary dormancy: seed longevity and secondary dormancy are two difference concepts, and affect different downstream target to affect seed dormancy, the author should also explain how DOG1 affect the seed longevity or seed lifetime. As for longevity, besides artificial simulation experiment, natural preservation for several month or yeast should be a better way, certainly, it needs long time for this experiment,

3) the author said that BRM affect asDOG1 antisense expression, thus the transgenic line expressing asDOG1 should be used to test their secondary seed dormancy or longevity

4) the effect of high temperature during secondary dormancy induction experiment or seed longevity on the expression of BRM and DOG1 should be measure, in order to their role in control seed germination, We speculate that high temperature did not affect the expression of BRM and DOG1 during this process.

5) except brm-3, more brm mutant should be used, two individual line should be better to explain one gene function, or its complement line with brm-3 genetic background. Similarly, the dominant line of dog1 should be used to explain the DOG1 function in secondary seed dormancy, not only use loss-of-function dog1-4 line.

Minor:
1) the phenotype brm-3: the author mentioned that BRM knockout allele brm-1 is sterile, so they therefore used the T-DNA insertion line brm-3, such line is norma or is week allele? itself show defective in seed germination or dormancy?

Dear Dr. Swiezewski:

Your manuscript has been reviewed by two experts in the field. The reviewers found your work intriguing, particularly noting the novelty of the proposed mechanism by which BRM controls DOG1 expression. However, both reviewers have highlighted several areas that require your attention for further improvement. Specifically, since the regulation through lncRNA asDOG1 transcripts is one of the major conclusions of this study. We encourage you to prioritize addressing comments related to the involvement of asDOG1 and to revise the manuscript to further substantiate this conclusion.

Warsaw 2024.10.11

Dear editor

Thank you for the opportunity to submit a revised version of our work. The attached point-by-point file includes a detailed response to each reviewer's questions. In agreement with your suggestion, we have focused on reviewers' points related to *asDOG1* regulation by BRM during secondary dormancy.

In the revised version of the manuscript, we have extended the FAIRE and ChIP-qPCR analysis to more data points along the secondary induction, included another BRM allele to support our claim, and added data for a transgene with antisense inactivated through TATA box mutations. Based on this we strongly believe all the points related to *asDOG1* and the vast majority of all other points raised have now been addressed.

Sincerely yours,

Dr Ruslan Yatusevich and Dr Szymon Swiezewski

Reviewer #1 (Comments for the Author):

This study revealed the involvement of BRM-associated SWI/SNF complex in the regulation of seed development and dormancy, and indicated that BRM regulates the process by cooperating with DOG1, a critical regulator of seed dormancy. The conclusion was supported by multiple methods, including genetic analysis, gene expression analysis, ChIP-PCR, and FAIRE. The study suggests a previously uncharacterized role in Arabidopsis, and demonstrates the underlying molecular mechanisms. However, I find that some experiments and analyses in the study need to be improved to confirm the conclusion.

We are grateful to reviewer for appreciating the novelty of our work and emphasising the diversity of the experiments we performed, as well as for their insightful analysis of our manuscript.

Major points:

1. The results shown in Figure 1B were not correctly described in the text (line 316-318). The results did compared the differentially expressed genes (DEGs) between brm-3 single mutant or dog1-4 single mutant and brm-3 dog1-4 double mutant but did not show a comparison of differentially expressed genes between the brm-3 and dog1-4 single mutants. Without the comparison of DEGs between brm-3 and dog1-4 single mutants, the relationship between the effects of brm-3 and dog1-4 on gene expression cannot be accurately evaluated.

As we write in the manuscript, most of the affected genes shared between *dog1-4* and *brm-3* mutants are also affected in the double mutant, as can be seen in columns 5 and 10 of the UpSet plot. A few genes shared between *dog1-4* and *brm-3* mutants but not affected in the double mutant are now included in the extended version of the UpSet plot (for example column 14). A comprehensive analysis of the overlaps between all single and double mutants is now provided and has been included in the revised manuscript as **Supplemental Figure 1C**. Initially, we did not present overlaps exclusive to single mutants, as there are only a limited number of such genes.

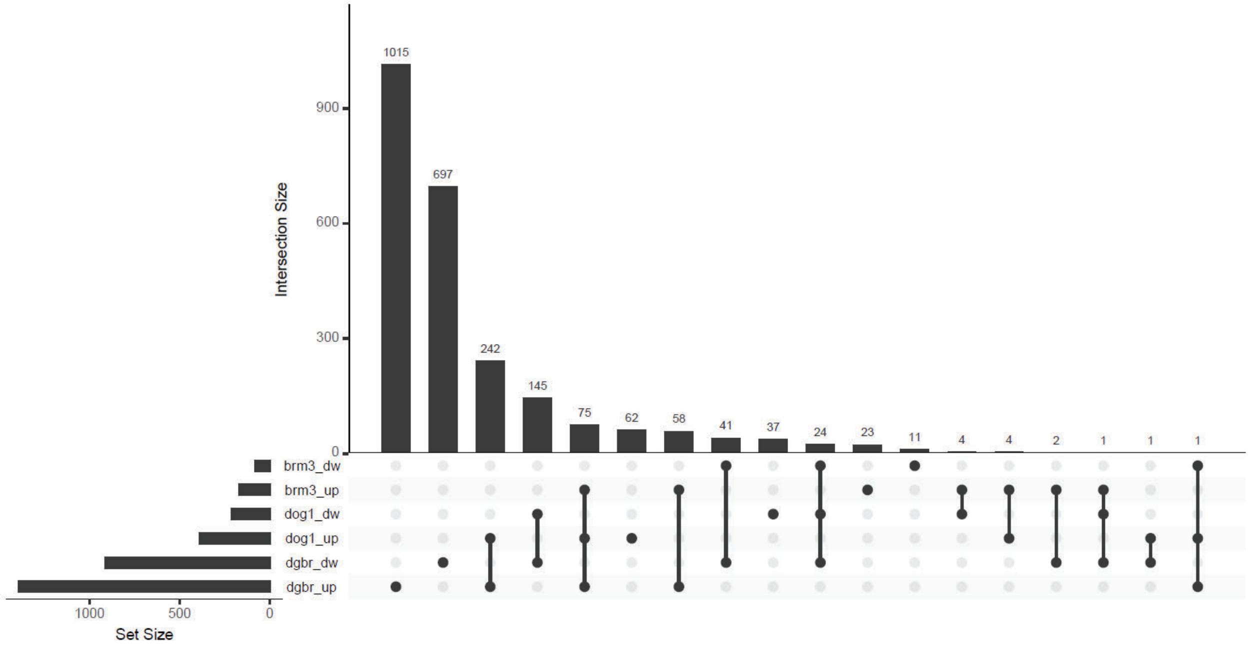

2. The clustering of gene expression profiles are shown in Figure 1C. The information of the number of genes is important for the analysis. Without this information, it is difficult to determine to what extent the opposite effects of brm-3 and dog1-4 occur at the whole-genome level.

We appreciate the Reviewer's valuable suggestion. In response, the revised **Figure 1C** now includes the number of genes in each cluster. Additionally, we have included the total number of genes categorised as DOG1-dependent and DOG1-independent in **Figure 1D**.

3. In line 343-346, related data described in the text should be indicated in the manuscript. Especially, the text indicated that 50 out of 244 genes are regulated by BRM in DOG1 dependent manner. This analysis was not shown.

The 55 genes out of the 244 DEGs represent the total number of DOG1-dependent cluster genes, as shown and described in **Figures 1C** and **1D**. We have now clarified the figure by including the number of genes in each cluster for **Figures 1C** and **1D**. Additionally, we have revised the figure legend to enhance clarity, particularly in relation to the cluster colour coding in **Figure 1D**.

4. The association of BRM with 3' end of DOG1 gene was indicated by ChIP-PCR. Because BRM ChIP-seq data have been shown in several previous study, whether the enrichment of BRM at the DOG1 locus is supported by these ChIP-seq data should be analyzed.

We have included the requested data for BRM and BRD ChIP-seq, as well as BRM ChIP-ChIP, in **Supplementary Figure 7A, B**. These data collectively support our conclusion that BRM and the SWI/SNF complex predominantly occupy the 3' end of the **DOG1** gene.

5. In Figure 3A and 3B, the direction of DOG1 gene and the position and direction of lncRNA within the DOG1 should be clearly marked. Otherwise, it is difficult to understand the results.

Thank you for bringing this to our attention. The issue has now been corrected.

6. By comparing Figure 3A and 3B, the authors suggest that the BRM binding position shifts from exon 2 and exon 3 regions to exon 3. Although the enrichment of BRM at the exon 3 is significant, I feel that the enrichment change at the exon 2 between the two conditions is not convincing. The error bars of the ChIP-PCR values at the exon1 and exon 2 regions as shown in Figure 3B are too high to differentiate the change between the two conditions.

We thank the Reviewer for highlighting this important issue. The results presented in **Figures 3A** and **3B** are derived from two independently conducted experiments, which is why there are notable differences in the % input scales, making direct comparisons problematic. In the revised version of the manuscript, we have avoided making direct comparisons and now focus solely on reporting significant enrichment for each specific time point.

7. In line 535, "BRM supresses pAsDOG1 activity". Within this this statement "BRM" should be corrected to "brm", because BRM can actually activate the expression of pAsDOG1.

This has now been corrected in the revised version of the manuscript.

8. While the increase of DNA accessibility was enhanced under 5 days of SD compared to 3 days of SD, it is unclear whether the increase is caused by the change of BRM enrichment between the two conditions. The experiment will further contribute to understanding of the underlying molecular mechanisms.

In the revised version, we provide a complete set of BRM ChIP-qPCR data for dry seeds as well as for 1, 3, and 5 days of secondary dormancy (SD) induction (**Figure 3A, B** and **Supplementary Figure 7C**). We also performed additional FAIRE analysis for dry seeds, 3, and 5 days of SD induction, which are now included in updated figures **(Figure 4A, B and Supplementary Figure 7D**). Please note that in the revised version of the manuscript, we have also normalised the FAIR data for unfair signal, allowing us to assess not only the differences between WT and *brm* but rather the generic accessibility profile. Based on this expanded dataset, we conclude that **BRM, while consistently bound to DOG1 in both dry seeds and during SD, remodels chromatin specifically during SD induction**, with chromatin accessibility gradually increasing between days 3 and 5.

9. Six positions were detected in FAIRE (Fig 4A, 4B), while seven positions were detected in ChIP-PCR (Fig 3A, 3B). Are the six positions used in FAIRE included in the seven positions used in ChIP-PCR? Because the FAIRE and ChIP-PCR data were compared in the text, this information should be clearly indicated.

The FAIRE experiment was specifically designed to analyse the transcription start sites (TSS) for both *DOG1* sense and antisense transcripts; therefore, we utilised a greater number of primers around the promoters and TSS for FAIRE. In the revised version of the manuscript in **Figures 3A, 3B; 4A, 4B** and **Supplementary Figure 7C, D,** we used the same number of regions/amplicons but for FAIRE they are concentrated around TSS while for ChIP they are focused around antisense start site. The list of primers used in these analyses is provided in **Supplementary Table 2**.

10. The different splicing forms of DOG1 mRNAs were detected in the study. The different forms need to be indicated by schemes in Figures. Moreover, the full names of shDOG1 and lgDOG1 need to be shown.

The full names of the short (*shDOG1*) and long (*lgDOG1*) forms of the *DOG1* gene have now been included in the revised text. Additionally, **Figure 4C, D and Supplementary Figure 15** have been updated to present a schematic representation of the *DOG1* gene, incorporating a detailed description of the *DOG1* variants.

Minor points:

1. In line 417, "that" should be corrected to "than".

This is corrected in the new version of the manuscript.

2. According to the text, the order of Figure 3E and 3F should be converted.

This is corrected in the new version of the manuscript.

Reviewer #2 (Comments for the Author):

BRM is putative component of SWI/SNF chromatin remodeling complex for gene epigenetic modification, and DOG1 is the main locus to determine primary seed dormancy, but their interaction was not full investigated before. In this manuscript, Wrona et al presented a series of subtle experiment to describe the novel function of BRM in control seeds quality and physiology, mainly in seed dormancy. Overall, this study is very interesting and expand our understanding on seed germination and dormancy process. Several concerns should be paid attention as below:

Main:

1) the mechanism of DOG1 in control secondary seed dormancy: it is known that DOG1 determine the primary seed dormancy, but the possible mechanism of DOG1 in control secondary seed dormancy is unknow, the author should provide more evidence and experiment to explain how DOG1 affect the seed dormancy, whether or how to affect ABA/GA level, starch metabolism or other possible pathway?

Thank you for this insightful question. Indeed, the precise mechanism of DOG1 involvement in secondary dormancy, including its relationship with ABA and GA levels and sensitivities, has not been fully clarified. We would like to emphasize that this manuscript was designed to explore the role of BRM and the SWI/SNF complex in the regulation of DOG1 through its antisense during secondary dormancy.

We strongly believe that a comprehensive exploration of DOG1's crosstalk with the ABA and GA pathways during secondary dormancy is beyond the scope of this work. However, we do provide data on the expression of GA and ABA marker genes during secondary dormancy in the *brm-3* mutant, and in the revised version, we include data for the same marker genes in the *dog1-4* mutant (Supplementary Figure 12).

In addition, the mechanism of secondary dormancy induction is only starting to be explored, but initial data from Botto lab showed that, in contrast to primary dormancy activation of GA catabolism is the main player in secondary dormancy induction. The same lab also showed that ABA and GA levels are not affected during secondary dormancy (Ibarra et al., 2016). Our RT-qPCR analysis of the marker genes in *dog1-4* and *brm-3* support this conclusion as we observed no changes in ABA marker genes but a significant change in GA catabolism marker genes in *brm3* mutant as well as some genes in *dog1-4* mutant (Supplementary Figure 12). Moreover, analysis of *DOG1* expression during SD in quadruple *nced2569* mutant (Supplementary Figure 10A) shows that *DOG1* expression and induction kinetics is unaffected by ABA suggested DOG1 regulation by BRM is independent from ABA level during secondary dormancy induction.

We hope this response satisfies the reviewer, as we believe our work paves the way for future analyses of plant hormones' involvement in DOG1-mediated secondary dormancy regulation.

2) the seed longevity and secondary dormancy: seed longevity and secondary dormancy are two difference concepts, and affect different downstream target to affect seed dormancy, the author should also explain how DOG1 affect the seed longevity or seed lifetime. As for longevity, besides artificial simulation experiment, natural preservation for several month or yeast should be a better way, certainly, it needs long time for this experiment,

Seed longevity and primary seed dormancy show a complicated relationship, with some researchers describing a positive and some negative correlation. It has been shown that the *dog1* loss-of-function mutant seeds have both reduced longevity and dormancy (Dekkers et al., 2016; Bentsink et al., 2006). We agree with the reviewer that artificial ageing is a proxy for natural ageing. In the revised version, we have included some preliminary data from naturally aged seeds stored for 4 years that show similar germination patterns to our artificially aged seeds (Supplementary Figure 4E).

3) the author said that BRM affect asDOG1 antisense expression, thus the transgenic line expressing asDOG1 should be used to test their secondary seed dormancy or longevity

We have previously published that *DOG1* antisense work in *cis* (Fedak et al 2016). For example, Figure 4B in (Fedak et al. 2016) show no *DOG1* expression change in pAS-DOG1 lines. Since *DOG1* antisense silences *DOG1* during primary and secondary dormancy, it is highly unlikely that a transgenic line expressing *asDOG1* alone will influence *DOG1* expression and secondary dormancy in *trans*.

4) the effect of high temperature during secondary dormancy induction experiment or seed longevity on the expression of BRM and DOG1 should be measure, in order to their role in control seed germination, We speculate that high temperature did not affect the expression of BRM and DOG1 during this process.

We are very grateful to the Reviewer for this valuable comment. We have now included data on *BRM* expression, as well as other elements of the SWI/SNF complex: *BRD1* and *SWP73A*, during the induction of secondary dormancy (Fig. 3H). *BRM* and *SWP73A* expression showed similar induction during secondary dormancy induction as *DOG1*. Interestingly, *BRD1*, which is one of 3 genes mutated in the triple *brd* mutant, showed no induction during secondary dormancy (Figure 3H). In addition, in the revised version of the manuscript, we show that BRM expression is unaffected by *dog1-4* while *DOG1* expression is induced in *brm-3* seeds (Supplementary Figure 10B).

5) except brm-3, more brm mutant should be used, two individual line should be better to explain one gene function, or its complement line with brm-3 genetic background. Similarly, the dominant line of dog1 should be used to explain the DOG1 function in secondary seed dormancy, not only use loss-of-function dog1-4 line.

As requested by the reviewer, we have included secondary dormancy data for another *brm* allele (Supplementary Figure 8B). The *brm-5* is a weak point mutation allele carrying G1137R substitution within the ATPase domain of BRM (Tang et al., 2008).

There is no perfect *DOG1* over expressor mutant. Here, we also included the data for *the LUC-DOG1 dTATA* transgenic lines (Supplementary Figure 13C). This line specifically inactivates the *DOG1as*. In agreement with the proposed role of *DOG1* antisense in secondary dormancy suppression, this lines showed a strong seed secondary dormancy phenotype (Supplementary Figure 13C).

Minor:

1) the phenotype brm-3: the author mentioned that BRM knockout allele brm-1 is sterile, so they therefore used the T-DNA insertion line brm-3, such line is norma or is week allele? itself show defective in seed germination or dormancy?

As described in the text *brm-3* is a weak allele. This mutant has a T-DNA insertion in the 3' part of the BRM gene, and the resulting BRM protein is shortened at the C terminus and shows only mild

developmental defects (Farrona et al., 2007, Archacki et al., 2013). As shown in the manuscript, the *brm-3* allele shows multiple defects in secondary dormancy and longevity but not in primary dormancy or seed germination. In addition, we have now included data from *brm-5* weak allele (Tang et al., 2008), to support our conclusions.

08-Nov-2024
Dr. Szymon Swiezewski
Instytut Biochemii i Biofizyki Polskiej Akademii Nauk
Warsaw 02-106
Poland

MSID: PP2024-RA-01076R1
MS TITLE: BRM-associated SWI/SNF complex controls Arabidopsis seeds quality and physiology

Dear Dr. Szymon Swiezewski:

I am pleased to inform you that your revised manuscript "BRM-associated SWI/SNF complex controls Arabidopsis seeds quality and physiology" has been accepted for publication in Plant Physiology. Your manuscript will be checked for consistency with journal formatting and image standards by one of our Science Editors, who will contact you shortly. They may have a number of queries and suggestions for you. Please keep in mind that they work to increase the online visibility of your article and its attraction for readers from the widest of backgrounds. You will have the opportunity to review any revisions, and I hope you will work constructively with them to get the most out of your research. You will also have the opportunity to review all changes when you receive the author proofs for your article.

Once validated for publication, your article will be sent to a Science Editor for a final review of your manuscript and source files. After your final files are uploaded, your article will appear in the next available issue and the online Preview version of your article will be posted after your signed Copyright and Checklist forms and final source files are received.

Please be sure to include the following in your manuscript file:
Harvard style citations
List of all author contributions and funding information at the beginning of manuscript after the titles and author list.
List all supplemental materials associated with the article, with titles, at the end of the ms, before the references and acknowledgments section.
Complete Figure Legends for all figures appearing in the article listed before the Literature Cited.

LICENSE INFORMATION Shortly after your paper is sent to production, you will receive an email from our publisher's author support team, **SciPris**, with information on article processing charges, as well as the types of Open Access license you would be able to purchase. The accepted version of your manuscript will not appear on our Advance Articles page until the license is selected and signed, so please look out for an email from SciPris. For more information see the Article Publication Fees section of our author guidelines: https://academic.oup.com/plcell/pages/General-Instructions

Finally, we encourage your submission of artwork for the journal cover. If there is an image or illustration related to your paper that you would like to have considered, please email it in .pdf or .tif format to clowe@aspb.org. An image title, brief, 2-3 sentence description of the image, and the name of the person credited for the image are also encouraged. If your image is selected to appear on the online cover of the journal, you will be contacted with the scheduled issue date and further information.

Thank you and congratulations on your Plant Physiology paper!

=====
FIRST AUTHOR PROFILES
=====

We recognize the hard work that goes into being First Author of a Plant Physiology paper by publishing a "First Author Feature" for each First Author alongside your paper.

If the first author(s) would like to submit information for a feature on Facebook and Twitter, fill out the form by clicking on the link below:

Plant Physiology First Author Feature Form: https://forms.office.com/r/ZBCGZfhm8F
If you cannot access the form through the link above, please contact Rachel at rbelsky@aspb.org.

To upload an image of yourself to accompany your profile, please put your photo in the correct journal folder at the link below.

You must label your image: Author First Name Last Name Article Title.

First Author Profile Image Dropbox: https://www.dropbox.com/scl/fo/xz029p7qjr2jml2rp2kmq/h?dl=0&rlkey=6fkxcaqmxx35ap152b2osy1fz

=====
IMPORTANT REMINDER: PEER REVIEW REPORTS
=====

If you opted to publish a peer review report along with your article during the original submission process, it will be prepared by the editorial staff and publicly posted with your manuscript, inside the zip file that contains any other supplemental material. As a reminder, the peer review report is a public record of all comments from editors and reviewers, as well as your prior responses, as you received them in the decision letters for each draft of your manuscript. If you agreed to publish this report and have changed your mind, or are not sure if you selected this option, please contact the editorial office as soon as possible before signing the license agreement from our publisher.

--- Reviewer comments:
Reviewer #1 (Comments for the Author):

My concerns have been addressed in the current version. The study reveals a previously unknown role of BRM in regulating seed development and dormancy, and illustrates the underlying mechanisms. I have no additional comments.

Reviewer #2 (Comments for the Author):

The authors already reply my questions, I have more inquiry for it, thank the authors for their efforts and nice work!

Sincerely,
Editor, Plant Physiology
